# A domesticated totivirus-like tandem array undergoes interspecific transfer and asymmetric evolution

**DOI:** 10.1093/molbev/msag269

**Published:** 2026-10-31

**Authors:** Derek J Taylor, Dominic A Tringali

**Affiliations:** Department of Biological Sciences, University at Buffalo, The State University of New York, Buffalo, NY, USA; Department of Biological Sciences, University at Buffalo, The State University of New York, Buffalo, NY, USA

**Keywords:** paleovirology, gene family, virus-host evolution

## Abstract

Functional genes derived from nonretroviral RNA viruses are generally expected to evolve more slowly following integration into host genomes. Here, we characterize *Scheffersomyces* totivirus-like open reading frame repeat module (STORM), a tandem array of four totivirus-derived ORFs (*TLC1* to *TLC4*) maintained in wood- and insect-associated yeasts for at least 15 My. STORM has nevertheless accumulated greater amino-acid divergence than sampled exogenous totiviruses. Host-calibrated rate analyses estimated that STORM evolved approximately 7-fold faster than related exogenous viral lineages. Evolution within the array was asymmetric. RELAX identified strong gene-wide relaxation in *TLC4* (*K* = 0.29), whereas BUSTED detected no episodic positive selection. Despite this relaxation, *TLC4* retained predicted decapping-associated residues, with evidence of purifying selection at several positions. Structural predictions indicated that STORM proteins retain a totivirus-like architecture, and RNA-seq comparisons revealed paralog-specific expression. Among 3,527 complete nuclear gene trees, none recovered all relationships defining any of the three STORM discordances with strong support, indicating that these patterns are exceptional relative to the nuclear genomic background. Local synteny and preservation of the four-copy array further support three interspecific transfers. In the best-supported transfer, STORM was flanked by an *ATP10* pseudogene and a transposase pseudogene from yeast endogenous element transposon (YEET), a newly identified Tc1/mariner-like transposon family. Both pseudogenes grouped phylogenetically with the inferred donor clade, and YEET1a-like terminal inverted repeats at the locus were characteristic of that clade. STORM therefore illustrates the selective maintenance, rapid asymmetric evolution, and horizontal mobility of a nonretroviral paleoviral array.

## Introduction

RNA viruses evolve rapidly over observable timescales, yet outbreak-calibrated molecular clocks often yield divergence times younger than minimum ages estimated from ancient paleoviral integrations (Duffy et al. 2008; Duchêne et al. 2014; Simmonds et al. 2019; Mifsud et al. 2025). The Prisoner of War framework attributes this time dependence to host-imposed constraints and sequence saturation, while allowing temporary acceleration when the selective environment changes, such as after a host switch (Ghafari et al. 2021, 2024). Tandem duplication after integration of viral genes into a host genome may also alter selective constraints. Here, we test whether these changes are accompanied by faster evolution relative to exogenous viral homologs.

Although ancient paleoviruses helped motivate time-dependent rate models, they remain underused as evolutionary systems. Paleoviruses arise when viral genetic material becomes integrated and retained in host genomes (Katzourakis and Gifford 2010; Patel et al. 2011). Most integrations are expected to decay as pseudogenes or be deleted unless maintained by selection at the host level. When recognizable endogenous and exogenous homologs are both available, paleoviruses provide a natural comparison between viral and host-genomic evolutionary regimes. They can also acquire host functions, establish minimum ages for evolutionary events, and reveal historical host ranges (Holmes 2011; Patel et al. 2011; Taylor et al. 2014; Ghosh et al. 2024; Barreat et al. 2025; Hu et al. 2025). Nonretroviral paleoviral loci can duplicate and form tandem arrays (Ballinger et al. 2014). Tandem duplication can increase expression and allow paralogs to retain, relax, or acquire different constraints, producing asymmetric divergence (Ohno 1970; Zhang 2003; Innan and Kondrashov 2010; Loehlin and Carroll 2016; Holland et al. 2017; Fan et al. 2023). Adjacent paleoviral copies are likely to descend from a single integration followed by duplication, making tandem arrays especially informative for studying post-integration rate changes.

Totiviruses are dsRNA viruses that primarily infect fungi, including yeasts and smut fungi, and are transmitted mainly through cell division, although phylogenetic evidence indicates occasional host jumps (Hughes et al. 2024). Totivirus-derived paleoviral sequences have undergone duplication in yeasts (Taylor and Bruenn 2009). They can also acquire antiviral functions: expression of a *Debaryomyces hansenii* capsid paleogene eliminated a related totivirus from *Saccharomyces cerevisiae*, whereas a translation-terminated construct did not (Warner et al. 2018). Yeast L-A and L-BC viruses modify their transcripts by capturing caps from host mRNAs (Fujimura and Esteban 2011). The resulting decapped host transcripts are exposed to Xrn1-mediated 5′-to-3′ degradation, while the superkiller RNA-surveillance (SKI) complex and cytoplasmic exosome restrict viral RNAs (Masison et al. 1995; Rowley et al. 2016). At controlled levels, L-A totiviruses can improve host fitness, whereas excessive viral accumulation can cause proteotoxic stress and reduce fitness (Chau et al. 2023; Hsiao et al. 2025). Their persistence therefore depends on interactions with host RNA-decay and virus-maintenance pathways.

A capsid-like array related to L-A totiviruses was first described in the genome of *Scheffersomyces stipitis* CBS 6054 (Taylor and Bruenn 2009). We designate this locus the *Scheffersomyces* totivirus-like open reading frame repeat module (STORM) and its four totivirus-like capsids *TLC1* to *TLC4*. The array is composed of four ORFs: a predicted capsid-RNA-dependent RNA polymerase (RdRp) polyprotein (*TLC2*) and three capsid-like paralogs. Previously published mass spectrometry detected a *TLC1* protein product in *S. stipitis* (Taylor et al. 2013). This variation in gene architecture suggests that the duplicates have experienced different selective constraints. However, earlier phylogenetic analyses based on a single STORM-containing genome did not resolve the placement of the *S. stipitis* capsid-like paleoviruses (Taylor and Bruenn 2009; Brejová et al. 2024).

Here, we investigate the tempo and mode of totivirus-like gene evolution across Serinales yeast genomes, focusing on wood- and insect-associated *Scheffersomyces* species whose shared microhabitats provide opportunities for interspecific contact (Shelomi 2025). We use “domestication” in its evolutionary-genomic sense to describe virus-derived loci that became stably integrated and selectively maintained rather than lost through neutral decay (Katzourakis and Gifford 2010; Henriques et al. 2024). We test three predictions. First, tandem duplication should permit asymmetric divergence and selection among *TLC* paralogs, potentially including accelerated evolution relative to sampled exogenous totiviruses. Second, selective preservation should be reflected in maintenance of the four-gene array, intact ORFs, conserved predicted protein structures, and transcription. Third, if STORM moved between species, its phylogeny and surrounding genomic context should conflict with the species tree, and the same relationships should be exceptional among nuclear gene trees.

## Results

### Totivirus-like tandem paleoviral arrays in *Scheffersomyces*

Genome screening of Serinales yeasts identified totivirus-like capsid and RdRp domains in multiple *Scheffersomyces* genomes, but only a subset carried the complete four-gene STORM array (Fig. 1a; Table S1). Array members generally retained conserved, intronless ORF boundaries across species. In contrast, *Scheffersomyces segobiensis* NRRL Y-11571, *Scheffersomyces quercinus*, *Scheffersomyces virginianus*, and *Scheffersomyces illinoinensis* retained only one or two fragmented, pseudogenized *TLC*-like remnants, consistent with independent degeneration of an ancestral array.

**Figure 1:**
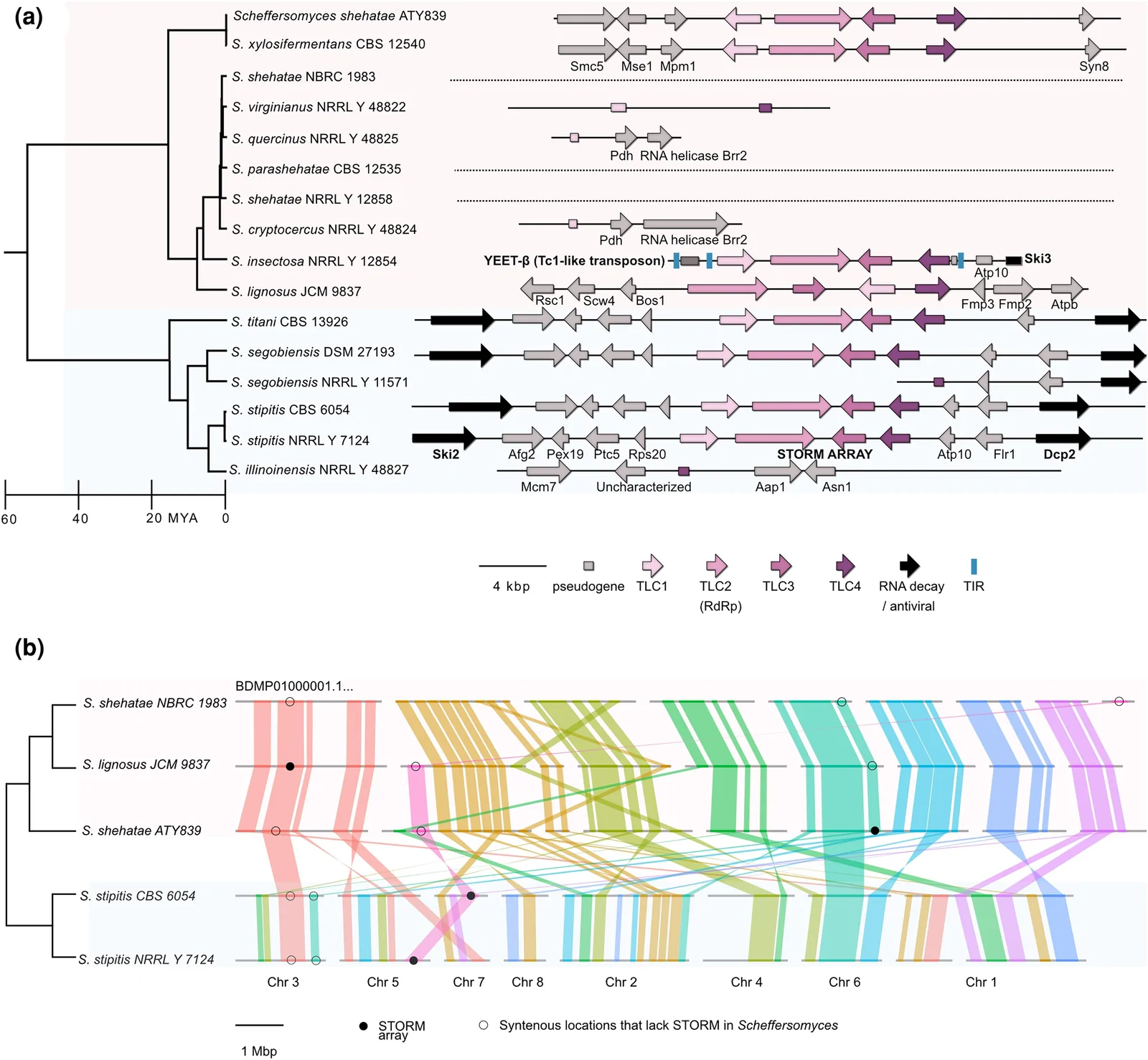
Synteny and genomic contexts of the STORM paleoviral array in *Scheffersomyces*. a) Left, chronogram for species of *Scheffersomyces* based on concatenated nuclear genes, with the time scale in millions of years before present (Ma), and right, gene neighborhoods surrounding the STORM array (colored blocks) mapped onto the tips of the tree; shaded rectangles represent the major clades of *Scheffersomyces* examined here. Arrows show ORFs and their transcriptional orientation; STORM components (*TLC1* to *TLC4* with *TLC2* encoding a capsid-RdRP-like polyprotein) are colored according to the legend (bottom). The architecture of a STORM-associated DNA transposon, YEET (Tc1-family), is shown for *S. insectosa*. Dotted lines mark genomes where a syntenic region is present, but the array has been lost or reduced to undetected fragments. b) Macrosynteny plot (ntSynt) showing whole-genome alignments for five genomes of *Scheffersomyces* ordered according to the species tree on the left (shaded colors represent two major clades of wood-digesting species). Each horizontal bar represents a chromosome or major scaffold, with colored blocks and ribbons connecting syntenic segments among genomes. Filled circles mark the positions of intact STORM arrays, whereas open circles indicate syntenic locations in other genomes. The bottom track shows the chromosome numbering for *S. stipitis* CBS 6054 (based on size), and the scale bar indicates 1 Mb.

### Long-term conservation and rapid loss of array architecture

Microsynteny analysis using flanking regions (10 to 15 kb on either side) showed that the four-gene architecture is conserved among *Scheffersomyces* species in which it occurs (Fig. 1a). Within the *titani*/*stipitis* clade, arrays occupy syntenic positions relative to neighboring host genes and maintain a conserved internal order (*TLC1* → *TLC2* [polyprotein] → *TLC3* → *TLC4*). In the *lignosus* clade, arrays retain a STORM-like architecture but occur in at least three distinct chromosomal contexts, suggesting translocation and/or horizontal transfer events (Fig. 1a and b). *Scheffersomyces lignosus* is the only species in our sampling in which *TLC1* occurs internally within the array.

Time-calibrated *Scheffersomyces* species trees based on concatenated nuclear genes place the common ancestor of the syntenic *titani*-clade array at more than 15 million years old (Fig. 1a), implying long-term persistence of the four-gene module. The minimum age may be much greater when the lignosus lineage is included: *S. lignosus* diverged from *Scheffersomyces titani* approximately 43.2 to 65.1 Ma, and each *S. lignosus* TLC paralog exceeds the total divergence observed across the syntenic *titani*-clade arrays.

STORM occurred in at least five distinct genomic contexts, including different locations among *S. stipitis* strains, and some arrays were subtelomeric. Despite extensive chromosomal rearrangements among deeper *Scheffersomyces* clades, the regions surrounding each STORM context remained locally syntenic across species (Fig. 1b). Within these homologous regions, a complete array was present in only one or a subset of genomes; genomes lacking it retained the corresponding flanks either without an intervening array or with decaying capsid-like fragments (Figure S1). We found no intact partial arrays; all detected partial arrays were pseudogenized.

### Association with antiviral, decapping, and mitochondrial genes

Across species, STORM shows recurrent associations with host genes implicated in RNA surveillance, antiviral defense, and mitochondrial function. In the *titani*/*stipitis* clade, STORM occurs between *SKI2*, encoding an antiviral helicase, and *DCP2*, encoding the major cytoplasmic decapping enzyme, separated by ∼13 kb and ∼5 kb, respectively (Fig. 1a). In this clade, the array is also flanked by *ATP10* and additional mitochondrial-associated genes (Fig. 1a). In *Scheffersomyces insectosa*, the antiviral gene *SKI3* occurs adjacent to the array; this array-adjacent copy is pseudogenized, but a second SKI3 copy outside the STORM-associated region remains intact. RELAX analyses of intact *SKI3* orthologs from STORM-containing wood-digesting *Scheffersomyces* clades detected significant relaxation relative to a reference clade [*K* = 0.75, likelihood ratio (LR) = 10.16, *P* < 0.001]. Relaxation was strongest in the *titani*/*stipitis* clade relative to the *lignosus*/*shehatae* clade (*K* = 0.14, LR = 175.26, *P* < 0.001). Likewise, *SKI2* and *DCP2*, which flank STORM in the *stipitis*/*titani* clade, also showed strong relaxation relative to the *lignosus*/*shehatae* clade. RELAX inferred near-neutralization for both genes when the *stipitis*/*titani* clade was designated as the test group (*SKI2*: *K* < 0.01, LR = 129.44, *P* < 0.001; *DCP2*: *K* = 0.07, LR = 33.90, *P* < 0.001).

### Genomic mobility and chimerism of STORM arrays

The amino-acid phylogeny, which included Serinales capsids together with all detected paleoviral and exogenous viral sequences, recovered a monophyletic STORM clade (SH-aLRT/UFBoot = 92.8/55) within the Serinales/L-A-like capsid group (Fig. 2). To test whether the divergent *TLC4* paralog destabilized the capsid phylogeny, we removed all 11 *TLC4* sequences and realigned. The reduced-taxon maximum-likelihood analysis then recovered a monophyletic *TLC1* to *TLC3* clade with increased support (SH-aLRT/UFBoot = 93.9/87). Thus, *TLC4* contributes to phylogenetic instability without altering the phylogenetic placement of STORM. A nucleotide phylogeny restricted to intact STORM ORFs (excluding the length-variable C-terminal region of approximately 60 amino acids) recovered the principal relationships found in the amino-acid tree, except that *TLC4* was sister to *TLC1* to *TLC3* (Fig. 3). Both analyses revealed multiple well-supported discordances with the *Scheffersomyces* species tree. For *TLC1 to TLC3*, sequences from *S. stipitis* NRRL Y-7124 grouped with *S. segobiensis* rather than with the conspecific strain *S. stipitis* CBS 6054; *TLC4* did not show this relationship. GHOST analyses recovered the two *S. stipitis* strains as sisters for *TLC4*, with strong support in five of six runs, while retaining the *S. segobiensis*/*S. stipitis* NRRL Y-7124 grouping for *TLC1 to TLC3*. In all analyses, STORM sequences from *Scheffersomyces xylosifermentans*, *Scheffersomyces shehatae*, and *S. insectosa* clustered within the titani/stipitis clade rather than with the lignosus/shehatae clade predicted by the species tree. The linked RdRp domain of *TLC2* recapitulated the two strongest local relationships within this discordance: *S. stipitis* NRRL Y-7124 grouped with *S. segobiensis* (SH-aLRT/UFBoot = 98.4/98), and the *S. xylosifermentans–S. shehatae* pair grouped with *S. insectosa* (98.6/89; Figure S2). This agreement indicates that the discordance is not restricted to the capsid alignment or driven solely by the divergent *TLC4* paralog. Separate approximately unbiased (AU) tests rejected the corresponding species-tree-constrained topology for every paralog (*P*_(_AU_)_ = 1.75 × 10^−8^, 1.03 × 10^−54^, 1.59 × 10^−4^, and 1.12 × 10^−4^ for *TLC1* to *LC4*, respectively; Table S3b). Thus, species-tree discordance was independently supported for all four paralogs, although the *S. stipitis* partial-replacement relationship was restricted to *TLC1* to *TLC3*. The recurrence of discordance across paralogs raised the possibility of horizontal gene transfer (HGT) involving the entire STORM array.

**Figure 2:**
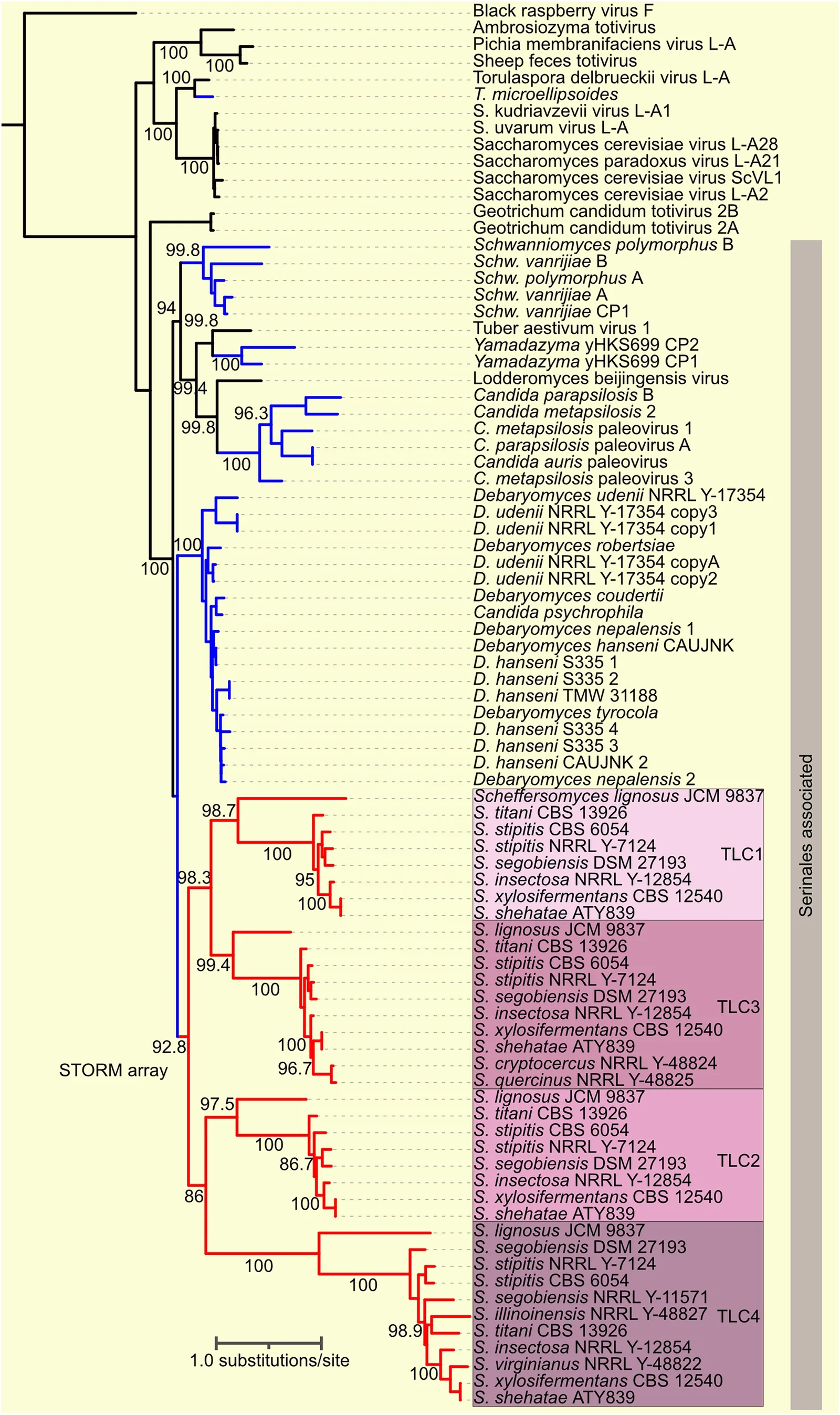
Amino-acid phylogeny of totivirus and totivirus-like capsid proteins. Maximum-likelihood phylogram inferred from amino-acid alignments of capsid-like domains. Nonitalicized names represent capsid sequences from exogenous totiviruses; italicized names correspond to totivirus-like paleoviruses. Labeled shaded rectangles highlight the STORM clade and its four capsid-like clades. Node labels show SH-aLRT support (%) for values ≥50. Sequence metadata are provided in Data S2.

**Figure 3:**
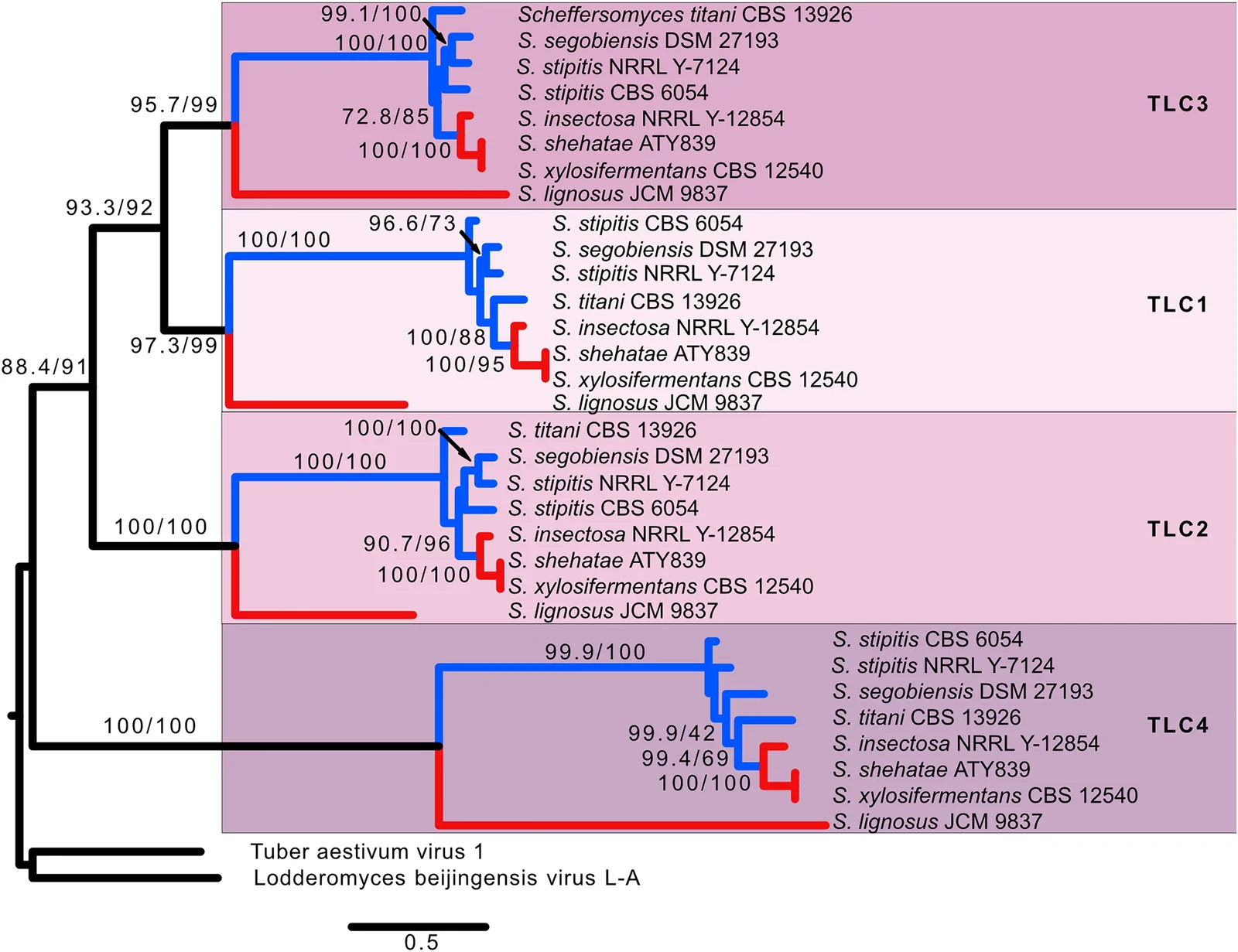
Nucleotide phylogeny of totivirus and totivirus-like capsid genes in *Scheffersomyces*. Maximum-likelihood tree inferred from codon-based alignments of capsid and capsid-like paleoviral ORFs. Shaded rectangles mark the four STORM capsid subclades (*TLC1* to *TLC4*) from species of *Scheffersomyces* (host genome and scaffold coordinates are given in Data S4). Red branches represent the lignosus clade, while blue branches indicate the titani clade, both from the species tree. The tree is rooted with capsid sequences from Tuber aestivum virus 1 and L. beijingensis virus L-A (unshaded). Node labels show SH-aLRT/ultrafast-bootstrap support (%) for values ≥50, and the scale bar denotes 0.5 nucleotide substitutions per site.

In *S. insectosa*, a pseudogene flanking the phylogenetically discordant STORM array showed highest sequence similarity to Tc1/mariner-like transposases from Mucorales fungi (phmmer: *Rhizopus arrhizus*, *E*-value 1.8 × 10^−93^) and highest structural similarity to a Tc1-like aspartate-aspartate-glutamate (DDE)-domain-containing protein from *Lichtheimia ramosa* (Foldseek vs. AFDB50, *E*-value 5.68 × 10^−32^). We designate this transposase family yeast endogenous element transposon (YEET; Figure S3). Phylogenetic analyses recovered two deeply divergent YEET lineages (YEET-α and YEET-β) whose host distributions were discordant with the species tree. Notably, YEET-β elements from *S. insectosa* (including the STORM-adjacent transposase), *Scheffersomyces cryptocercus*, and *S. shehatae* were nested within a clade dominated by *S. stipitis*, *S. segobiensis*, and *S. titani*. These discordant YEET elements showed little sequence divergence from their relatives in the *S. stipitis/S. segobiensis/S. titani* clade, consistent with recent transfers. At the *S. insectosa* STORM locus, the associated terminal inverted repeats (TIRs) are of the YEET1a type. This subtype is common in the *S. segobiensis/S. stipitis/S. titani* group and otherwise occurs in the lignosus/shehatae clade only among phylogenetically discordant YEET elements. The TIR subtype therefore provides an additional donor-lineage signature at the recipient locus. Overall, we identified at least five YEET discordances consistent with horizontal transfer among rotten-wood-associated *Scheffersomyces* species (Figure S3).

The STORM-containing locus in *S. insectosa* showed contrasting phylogenetic affinities across its flanking regions. The STORM-adjacent *SKI3* pseudogene grouped with the intact *S. insectosa SKI3* copy with maximal support (SH-aLRT/UFBoot = 100/100; Figure S4a), consistent with in situ pseudogenization. In contrast, the STORM-linked *ATP10* pseudogene grouped within the *S. segobiensis/S. stipitis/S. titani*-associated clade (Figure S4b) rather than with the intact *S. insectosa ATP10*. This affinity is not observed in the species tree or other protein-coding gene phylogenies (see below), indicating that it is specific to STORM and its flanking region. These results support a mosaic locus in *S. insectosa*: YEET, STORM, and *ATP10* show titani/stipitis-clade affinities, whereas the flanking *SKI3* pseudogene is most closely related to an intact *SKI3* copy in *S. insectosa*.

Sequence divergence provides additional support for a shared history of this genomic region. Between *S. insectosa* and *S. segobiensis*, K2P distances were similar for *ATP10* (0.310), YEET1a-flanking sequence (0.285), and YEET1a fragments (0.246), and were lower than the approximately 0.45 distance at TLC third-codon positions. Although *ATP10* lies outside the TIR-bracketed interval, its phylogenetic affinity and physical linkage provide evidence that the surrounding region shares the same unusual evolutionary history. Low divergence among *S. insectosa* YEET transposases (mean K2P ≈ 0.053) further indicates recent YEET activity. An initial survey of 272 nuclear genes showed that the STORM-like topology (based on nucleotides) was rare, and STORM patristic distances exceeded the reference-gene distribution (exact Mann–Whitney *P* = 1.53 × 10^−10^; Fig. 4a). These results support an unusual, locus-specific evolutionary history. Two TIR-like sequences bracket the STORM-containing interval, a third degenerate repeat lies upstream, and one boundary is shared with the adjacent YEET1a element. This complex organization is consistent with sequential or nested YEET activity followed by erosion. No unambiguous two-base-pair 5′-TA-3′ target-site duplication was detected around the complete interval. TIR types, YEET subfamilies, and element counts are provided in Data S1 and Table S2.

**Figure 4:**
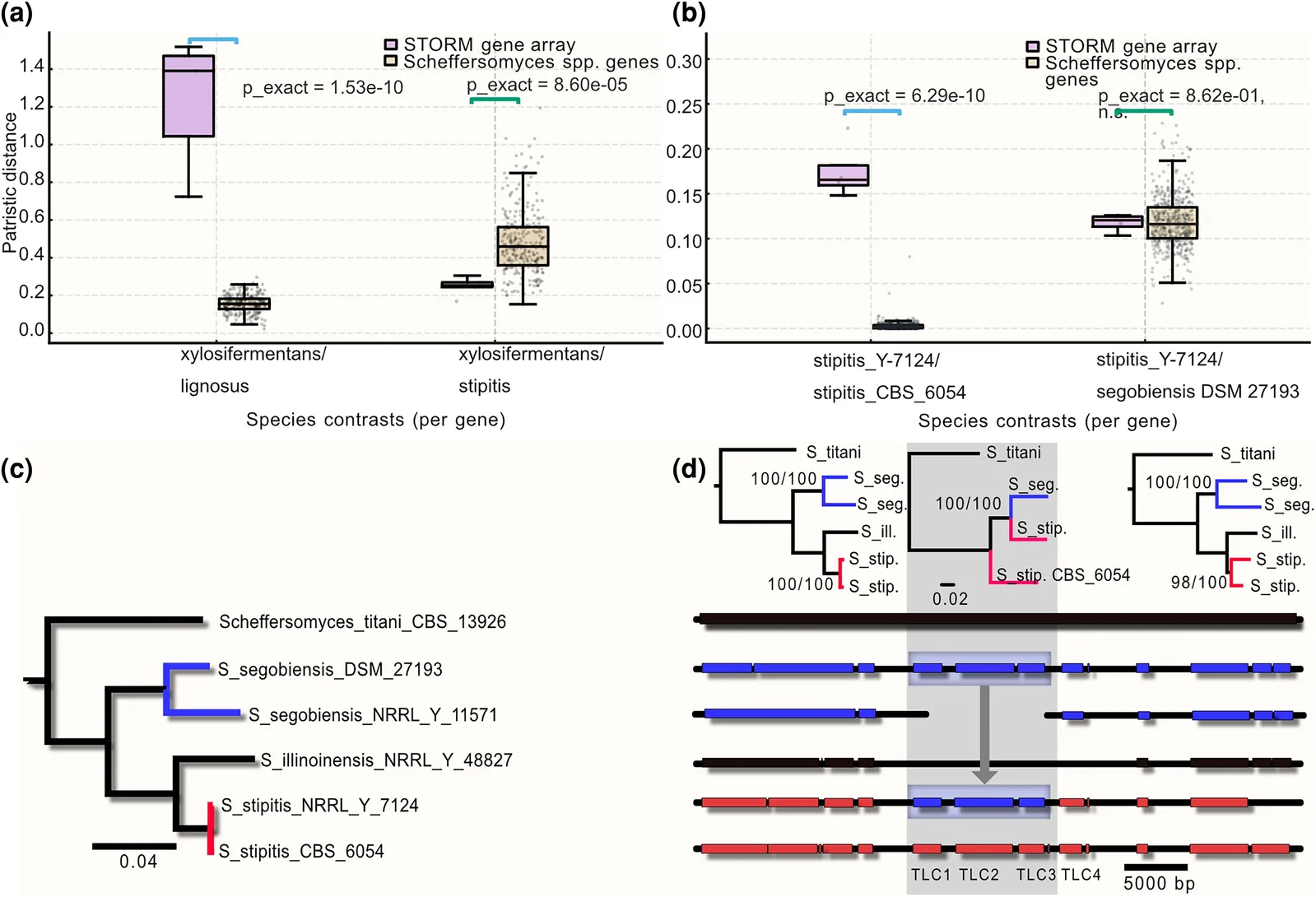
Reticulate transfer of the STORM array inferred from patristic distances and flanking-region phylogenies. a) Patristic distances between *S. xylosifermentans* and *S. lignosus* (left) and between *S. xylosifermentans* and *S. stipitis* CBS 6054 (right), comparing the STORM array (purple box plots) with 272 reference nuclear genes from *Scheffersomyces* (beige box plots). b) Patristic distances between *S. stipitis* NRRL Y-7124 and *S. stipitis* CBS 6054 (left) and between *S. stipitis* NRRL Y-7124 and *S. segobiensis* DSM 27193 (right). c) Species tree for the *titani/stipitis/segobiensis* clade based on concatenated nuclear genes, showing that the two *S. stipitis* strains (red branches) form a monophyletic group, with monophyletic *S. segobiensis* (blue branches) more distantly related. d) Phylogenies of genomic regions flanking the STORM array (upper panels) and corresponding BLASTn similarity blocks using the *S. titani* region (JAJMBD010000002.1:430000-519758) as a query (lower panel). Trees from upstream and downstream segments recover the expected species relationships, with *S. stipitis* strains forming a clade and *S. segobiensis* outside (blue vs. red bars), whereas the array region itself (gray block) is discordant. The gray arrow represents the proposed direction of gene transfer.

For *S. stipitis* strains, only one of 523 reference-gene nucleotide alignments showed the STORM-like discordance, whereas *TLC1* to *TLC3* divergence between conspecific *S. stipitis* strains exceeded all reference-gene distances (Fig. 4b). The species tree for the *titani*/*stipitis*/*segobiensis* clade places the two *S. stipitis* strains as monophyletic sister taxa with monophyletic *S. segobiensis* as outgroup (Fig. 4c). Trees from regions upstream and downstream of *TLC1* to *TLC3* recover this expected species topology with strong bootstrap support (Fig. 4d), but the tree from *TLC1* to *TLC3* itself places *S. stipitis* NRRL Y-7124 with *S. segobiensis* rather than with conspecific *S. stipitis* CBS 6054. There is a sharp topological transition at the *TLC1* to *TLC3* boundaries, consistent with a localized transfer event that replaced the STORM region of NRRL Y-7124 while leaving the surrounding genomic context intact. This partial-replacement pattern, with the *TLC3* to *TLC4* junction marking an apparent internal breakpoint, is consistent with recombination between donor and recipient arrays during the transfer event.

We expanded the discordance analysis using STORM-excluded proteomes from 18 taxa. OrthoFinder identified 3,689 primary single-copy gene trees, including 3,527 containing all taxa. ASTRAL recovered the same unrooted species topology from both datasets, regardless of whether weakly supported gene-tree edges were collapsed. This topology also agreed with our 10-gene tree and that of Opulente et al. (2024).

Full-tree analysis of the 3,527 complete 18-taxon nuclear gene trees found no tree that reproduced all strongly supported relationships defining HGT1, HGT2, or HGT3 (Table S3; Data S10). Three trees recovered one of the two nested relationships required for HGT2, but none recovered its complete topology; no complete or partial match occurred for HGT1 or HGT3. An *S. insectosa*-only test likewise recovered no supported HGT1 node. Thus, all three STORM discordances were exceptional relative to the nuclear genomic background.

### Asymmetric divergence and accelerated STORM evolution

We next compared accumulated amino-acid divergence among extant L-A-like totiviruses, totivirus-like paleoviruses outside STORM, and the four STORM paralogs. In the maximum-likelihood capsid tree, root-to-tip distances were greatest for *TLC4* (mean 5.05 expected amino-acid substitutions per site; Fig. 5a; Table S4), intermediate for *TLC2* and *TLC3* (approximately 3.8 to 3.9), and lowest within STORM for *TLC1* (3.66). L-A-like totiviruses were more divergent than non-STORM paleoviruses but less divergent than STORM paralogs. All pairwise group contrasts except *TLC2* vs. *TLC3* were significant after the false discovery rate (FDR) correction (Table S5). ClipKIT filtering removed 25.4% of sites but retained the relative pattern (Figure S5), and within-group pairwise distances likewise separated STORM from exogenous viruses and non-STORM paleoviruses (Fig. 5b). RdRp-domain comparisons, for which STORM was represented by *TLC2*, showed a similar pattern of accumulated divergence (Fig. 5c; Figure S2). Removing the non-STORM paleoviruses from the capsid-like alignment resulted in an alternative topology grouping the sampled exogenous viruses together. However, the RdRp phylogeny agreed with the full capsid-like tree placing STORM with the *Lodderomyces beijingensis* virus*/*Tuber aestivum virus lineage. To compare rates and assess the influence of topology, we evaluated the main and alternative topologies using the same matched 46-sequence capsid alignment in host-time-constrained phylogenetic analysis by maximum likelihood (PAML) local-clock analyses under Q.YEAST+Γ4. We fixed the STORM crown at 43.2, 54.0, or 65.1 Ma and the node joining Torulaspora delbrueckii virus with the sampled Saccharomyces L-A virus clade at 97 Ma. At the central STORM age of 54 Ma, the estimated STORM/background rate ratios were 6.98 under the main topology and 7.05 under the alternative topology. Across the tested STORM ages, these ratios ranged from 5.75 to 8.79 and from 5.78 to 8.93, respectively (minimum 2Δln *L* = 998.92; all nominal *P* < 10^−218^). Replacing the 97-Ma constraint with a deeper host-based Lodderomyces-Saccharomyces calibration (198.5 to 277.6 Ma) also supported faster STORM evolution under both topologies, with rate ratios of 7.44 and 7.59 at the central calibrations (Table S6).

**Figure 5:**
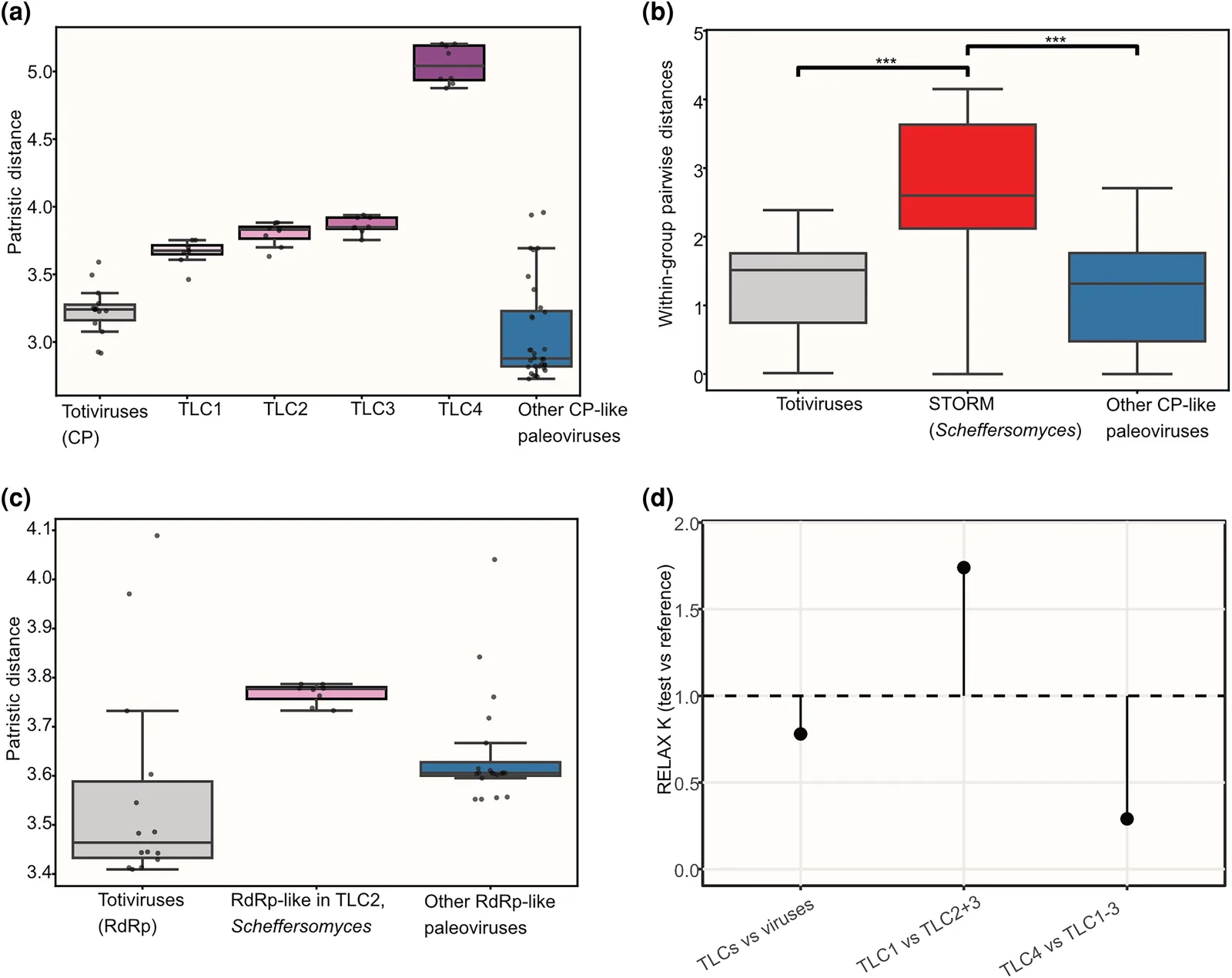
Amino-acid divergence and selection-intensity variation in STORM capsids and RdRp. a) Root-to-tip patristic distances for CP amino-acid trees, showing exogenous totiviruses (gray box plots), the four STORM copies *TLC1* to *TLC4* (plum box plots), and other totivirus-like capsid paleoviruses (blue box plots). b) Within-group pairwise patristic distances for capsid sequences, comparing exogenous totiviruses (gray box plot), STORM capsids from *Scheffersomyces* (red box plot), and other capsid paleoviruses (blue box plot). c) Root-to-tip patristic distances for RdRp trees, comparing exogenous totiviruses (gray box plot), the RdRp-like paleovirus from *Scheffersomyces* STORM (plum box plot), and other RdRp-like paleoviruses (blue box plot). d) RELAX tests of selection intensity (K) for capsid genes. From left to right: all STORM *TLCs* vs. exogenous viruses; *TLC1* vs. *TLC2*/*TLC3*; and *TLC4* vs. *TLC1* to *TLC3*. *K* < 1 indicates relaxation in the test set; *K* > 1 indicates intensification. The dashed line marks *K* = 1.

### Relaxed selection and structural evolution of a divergent copy

To determine whether the elevated divergence of *TLC4* reflected weakened purifying selection, episodic positive selection, or site-specific differences in constraint, we applied several codon-based tests (Fig. 5d; Table S7). RELAX indicated no statistically significant evidence of relaxation across STORM capsid branches relative to exogenous viral capsids (*K* = 0.78, LR = 3.16, *P* = 0.075). Within STORM, however, *TLC4* showed strong evidence of relaxed selection relative to the remaining *TLC* paralogs (*K* = 0.29, LR = 74.13, *P* < 0.001). Under the RELAX alternative model, the nonzero purifying selection rate class shifted from ω = 0.090 on *TLC1* to *TLC3* reference branches to ω = 0.497 on TLC4 test branches. By contrast, *TLC1* exhibited intensified selection relative to *TLC2* and *TLC3* (*K* = 1.74, LR = 16.47, *P* < 0.001). BUSTED provided no evidence that the *TLC4* pattern resulted from episodic diversifying positive selection. The *TLC4*-only gene-wide test was nonsignificant (*P* = 0.50), and no positively selected ω class had nonzero weight in the alternative model. Thus, *TLC4* is better characterized as evolving under relaxed purifying selection than recurrent positive selection. Contrast-fixed effects likelihood (FEL) showed that *TLC4* has a distinct selective regime among paralogs. 24 *TLC1*-*TLC4* and 28 *TLC2*/3-*TLC4* differences remained significant at the exploratory FDR threshold of *q* ≤ 0.20, whereas no *TLC1* to *TLC2*/3 difference remained significant. In the separate foreground-background analysis, 43 sites had higher nonsynonymous rates on *TLC4* branches. In the context of the RELAX and BUSTED results, this pattern is consistent with localized weakening of constraint. None overlapped the decapping-associated positions, although two sites immediately preceded the loop (Figure S6).

We next used FUBAR Approach to Directional Evolution (FADE) to test whether *TLC4* branches showed repeated substitutions toward particular amino acids. Two sites outside the predicted decapping-related regions showed evidence of directional evolution: S4 position 63 toward glycine (Bayes factor = 106.49; three inferred Ala→Gly substitutions) and position 165 toward proline (Bayes factor = 113.84; three inferred Ser→Pro substitutions). At the YHDGH-related loop and downstream Gly-Tyr (GY) and Phe–Ala–Pro (FAP) positions, Bayes factors (BFs) ranged from only 1.2 to 1.7, far below the prespecified threshold of 100.

FEL analysis of the eight intact *TLC4* sequences indicated that purifying constraint persisted at some sites despite the gene-wide relaxation detected by RELAX. Across all 648 codons, 22 sites showed evidence of purifying selection after alignment-wide Benjamini–Hochberg (BH) correction at *q* ≤ 0.10, including seven at *q* ≤ 0.05; no site showed diversifying selection after this correction. Of the seven decapping-associated positions defined a priori from independent viral evidence, two were invariant at the nucleotide level and therefore uninformative for FEL. None of the remaining five passed alignment-wide correction, but four showed evidence of purifying selection after Benjamini–Hochberg correction within the targeted five-site set at *q* ≤ 0.10, including one at *q* ≤ 0.05 (Figure S6; Table S8).

Within this same *TLC4*-only FEL analysis, site-specific constraint at the decapping-associated loop was examined in more detail. Among the sampled extant totiviruses, the homologous loop occurs in three forms: YHDGH, YDDGH, and YNDGH (Fig. 6). *TLC4* retains two of these viral forms (YNDGH and YDDGH) in the sampled genomes. The loop Y, G, and terminal H positions showed only synonymous variation, with β estimates of approximately zero. Their raw *P*-values were 0.0666, 0.00443, and 0.0472, respectively, and their targeted *q*-values were 0.0941, 0.0221, and 0.0941. The second loop position, occupied by N or D in *TLC4*, showed no significant evidence of selection (β = 0.108; raw *P* = 0.5981; targeted *q* = 0.5981). The central aspartate residue was encoded by the invariant codon GAC in all eight intact TLC4 sequences and was therefore uninformative for FEL. All three recent *TLC4* pseudogenes also retained their corresponding loop sequence but were excluded from FEL because their terminal branches no longer represent intact coding sequences.

**Figure 6:**
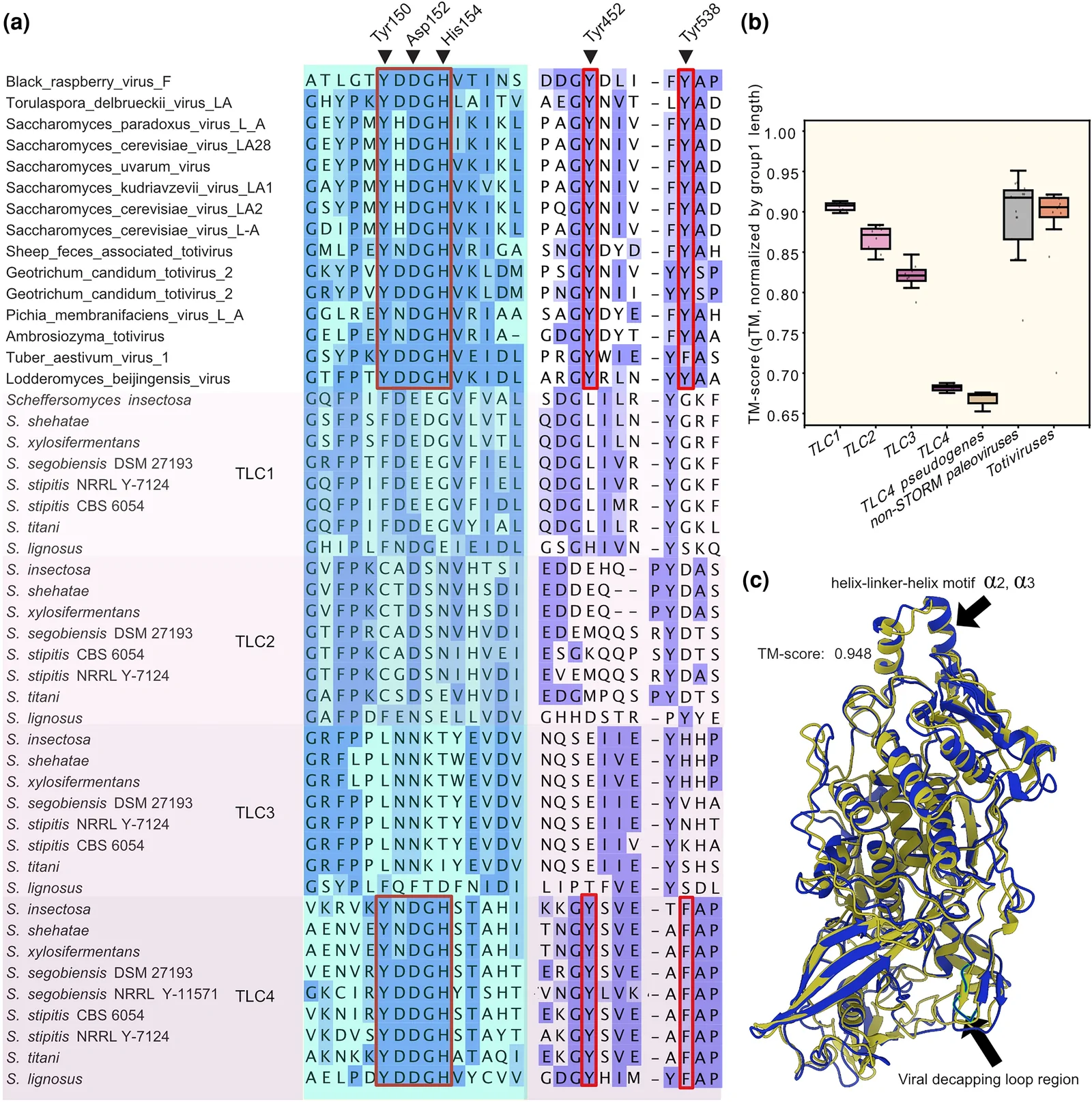
Conservation of the decapping-loop motif and local capsid structure in STORM paleoviruses. a) Amino-acid alignment of the region encompassing the putative decapping loop in totivirus capsid proteins and totivirus-like capsids from *Scheffersomyces* and other yeasts. Viral capsids are shown at the top, followed by the four STORM copies (*TLC1* to *TLC4*; shaded background). Conserved residues implicated in decapping activity are highlighted by red boxes. b) qTM-scores (normalized by viral query length) between AlphaFold paleoviral capsid predictions and exogenous totivirus references. Each paleoviral structure was assigned the highest TM-score obtained against any Serinales-associated viral reference. Non-STORM TLCs were filtered to retain only high-coverage alignments (query coverage ≥80%; 32 → 17 structures). Group differences were significant overall (Kruskal–Wallis, *P* < 0.001), with *TLC4* ORFs showing significantly lower structural similarity to viral capsids than *TLC1* and non-STORM paleoviruses (pairwise Mann–Whitney tests; see Data S7). Mean predicted local distance difference test (pLDDT structure confidence score) was similar for *TLC4* ORFs (83.4), *TLC2* (83.7), and *TLC3* (84.1), indicating that lower *TLC4* qTM-scores were not explained by lower prediction confidence (Figure S7). c) Representative AlphaFold3-predicted structural overlay of *TLC1* (*S. stipitis*; gold ribbon) with an experimentally determined totivirus capsid reference, Saccharomyces cerevisiae L-A virus (blue ribbon; PDB 8A5T).

*TLC1*/2/3 showed a different pattern. Positions 154 and 155 were not detectably constrained, whereas positions 153, 156, and 157 showed purifying selection on paralog-specific residue states rather than preservation of the complete ancestral viral motif (targeted *q* = 0.0061, 0.0292, and 0.0667, respectively). A similar asymmetry occurred at the downstream structural positions. The tyrosine in the *TLC4* GY element at S4 position 460 was retained through synonymous codon variation (*β* ≈ 0, *P* = 0.0753, targeted *q* = 0.0941), whereas *TLC1*/2/3 showed purifying selection on their respective non-tyrosine states (*β* = 0.377, *P* = 0.00152, targeted *q* = 0.0061). The phenylalanine in the *TLC4* FAP element at position 564 was invariant as TTT, whereas the homologous position was more variable in *TLC1*/2/3 (*β* = 0.253, *P* = 0.1055, targeted *q* = 0.1477). Together, these analyses show that *TLC4* combines strong gene-wide relaxation with localized constraint at several prespecified decapping-associated positions, rather than evolving as a uniformly unconstrained pseudogene.

GARD found no significant evidence of recombination in the *TLC4* alignment. The recombination model did not fit better than the single-tree model [corrected Akaike information criterion (AICc) = 17,926.95 vs. 17,926.63], and support for potential breakpoints was weak (maximum = 0.41; no site exceeded 0.50).

To assess the structural consequences of *TLC4* divergence, we predicted capsid structures for 80 viral and paleoviral sequences representing seven structural groups using AlphaFold3 and compared them with exogenous totivirus references using Foldseek (Abramson et al. 2024; van Kempen et al. 2024; Data S7). Prediction confidence was high across all groups: group mean pLDDT scores ranged from 82.3 for *TLC4* pseudogenes to 87.8 for exogenous totiviruses, and at least 84% of residues in each group exceeded pLDDT = 70. *TLC4* ORFs had a mean pLDDT of 83.4 (individual means 79.4 to 86.1), with 84.0% of residues exceeding 70, closely matching *TLC2* (83.7; 84.2%) and *TLC3* (84.1; 85.6%). Group-level pLDDT distributions are provided in Figure S7. Predicted aligned error (PAE) heatmaps for a representative structure from each group and for all eight *TLC4* ORFs are provided in Figures S8 and S9, respectively. TLC4 therefore showed comparable overall prediction confidence to TLC2 and TLC3 despite its lower structural-similarity scores.

Foldseek comparisons using query-normalized TM-scores showed that *TLC4* ORFs retain a recognizable totivirus-like capsid fold despite high sequence divergence (Fig. 6b; Data S7). Exogenous totiviruses had the highest within-group structural similarity, while *TLC1* and singleton paleoviruses showed the greatest similarity to exogenous viral references. *TLC2* and *TLC3* were intermediate. *TLC4* ORFs had significantly lower TM-scores than *TLC1* in FDR-corrected comparisons but remained more similar to viral capsids than *TLC4* pseudogenes. Because *TLC4*, *TLC2*, and *TLC3* had comparable pLDDT distributions, the TM-score differences are unlikely to arise primarily from differential prediction confidence.

Inspection of the predicted structures localized much of this divergence to surface loops and protruding domains. *TLC1* to *TLC3* retained the core totivirus-like β-sandwich and surrounding α-helices but lacked the predicted decapping-associated residues (Fig. 6a). Direct comparison with an experimental L-A capsid structure confirmed strong structural similarity to STORM across all four paralogs (Fig. 6c; Figure S10). *TLC4* retained the YDDGH/YNDGH loop and downstream GY and FAP residues together with the predicted loop configuration, but it diverged more in other surface-exposed regions and lacked the conserved totiviral α2-α3 helix-linker-helix feature (Okamoto et al. 2020; Grybchuk et al. 2022; Fig. 6c; Figure S10). Thus, *TLC4* retains a core capsid-like fold and predicted decapping-associated architecture while showing localized structural divergence.

### STORM transcription and condition-associated expression

To assess STORM transcription, we quantified RNA-seq reads across five *Scheffersomyces* species/strain datasets (see Methods). Transcripts from all four *TLC* loci were detected, with *TLC1* generally the most abundant (Fig. 7a). Descriptive within-sample expression was scaled to the geometric mean of five candidate reference genes, and an *ACT1*-only sensitivity analysis produced highly concordant contrasts (Pearson *r* = 0.990; maximum absolute difference = 0.149 log_2_-fold-change units; Figure S11). Differential-expression inference was restricted to replicated comparisons: four biological replicates per condition for *S. xylosifermentans* and two per condition for *S. stipitis* CBS 6054. Unreplicated datasets, including *S. insectosa*, were used only as descriptive evidence of transcription.

**Figure 7:**
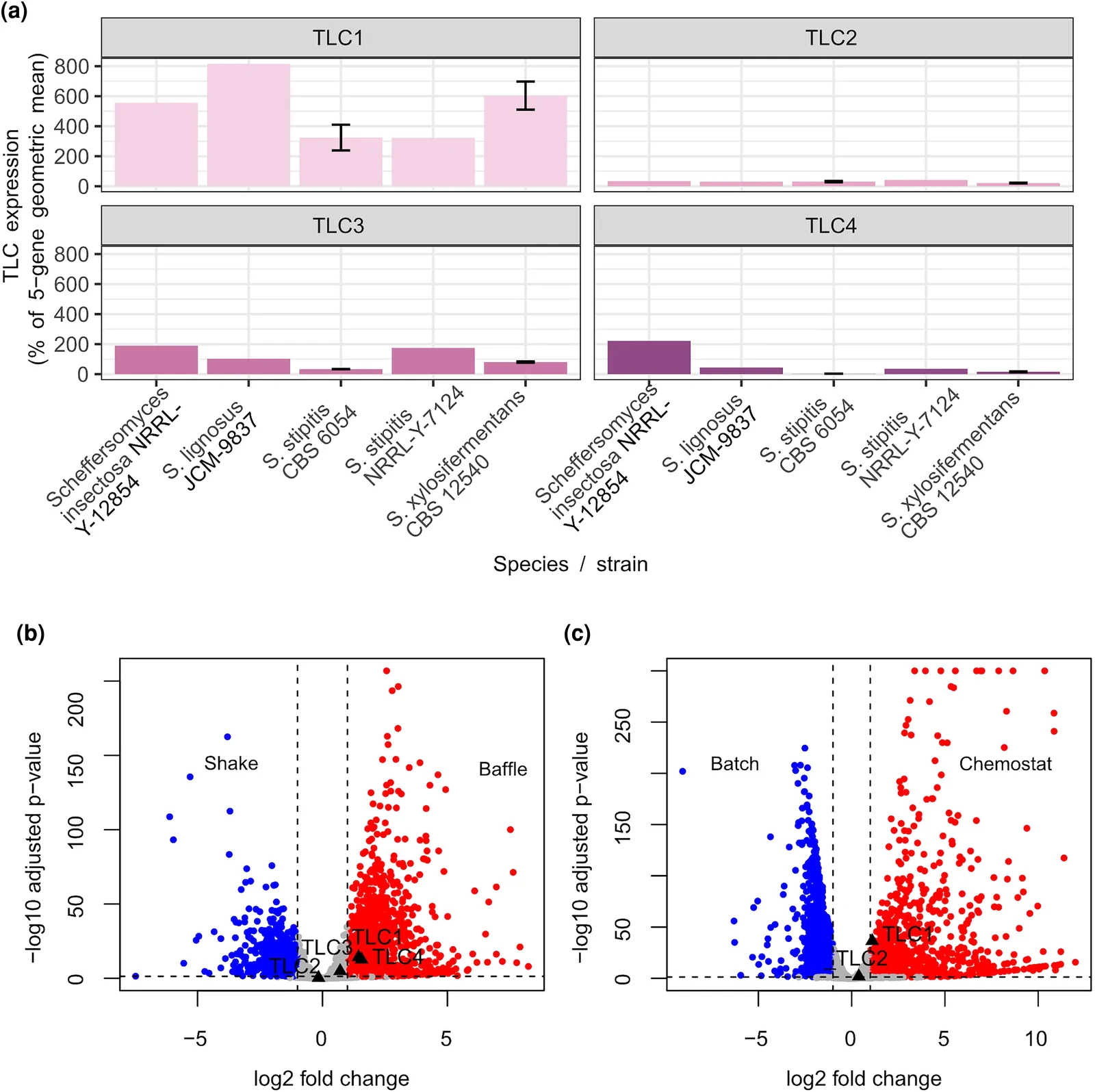
Cross-species expression of STORM capsid genes in *Scheffersomyces*. a) Bar plots show mean expression of *TLC1* to *TLC4* across five *Scheffersomyces* species and strains, expressed as a percentage of the geometric mean of five candidate reference genes. Error bars indicate ± SE where biological replicates were available; single-sample datasets are descriptive only. b) Differential expression of STORM capsids in *Scheffersomyces* across growth regimes. Volcano plot for *S. xylosifermentans* comparing baffle (higher O_2_ transfer) vs. shake cultures. Each point represents a gene; the *x*-axis shows log_2_-fold change (positive values = higher expression in baffle, negative = higher in shake), and the *y*-axis shows −log_10_-adjusted *P*-value. Red and blue points indicate significantly upregulated genes in baffle and shake, respectively (FDR threshold indicated by the horizontal dashed line; vertical dashed lines mark fold-change cutoffs). Triangles highlight the four STORM capsid genes (*TLC1* to *TLC4*). c) Volcano plot for *S. stipitis* CBS 6054 comparing chemostat vs. batch cultures, plotted as in (b); positive values indicate higher expression in chemostat.

In *S. xylosifermentans*, *TLC1* and *TLC4* expression was higher in high-aeration baffled flasks than in moderately aerated shake flasks (Fig. 7b). *TLC1* increased 2.96-fold (log_2_FC = 1.566, FDR = 2.38 × 10^−13^), and *TLC4* increased 2.72-fold (log_2_FC = 1.444, FDR = 5.46 × 10^−15^); both exceeded the twofold effect-size threshold. *TLC3* increased by 1.63-fold (log_2_FC = 0.708, FDR = 1.32 × 10^−5^), and *TLC2* did not change significantly (log_2_FC = −0.158, FDR = 0.462). Thus, the largest expression differences between the two aeration conditions occurred in *TLC1* and *TLC4*. In *S. stipitis* CBS 6054, *TLC1* expression was 2.15-fold higher in chemostat than in batch cultures (log_2_FC = 1.101, FDR = 3.43 × 10^−37^). *TLC2* increased by 1.30-fold (log_2_FC = 0.383, FDR = 0.0127), which was statistically significant but below the twofold threshold (Fig. 7c). *TLC3* and *TLC4* changed little. Several candidate host genes also differed significantly between conditions, but all changes were less than 2-fold (|log_2_FC| < 1). Their expression changes showed no consistent correspondence with those of the TLC loci across the examined datasets (Figure S12a and b).

## Discussion

### Tandem paleoviral arrays as evolutionary modules

STORM is a four-copy tandem paleoviral array whose paralogs have evolved under distinct selective regimes while retaining totivirus-like structural features. Its architecture has persisted for at least 15 My within the syntenic titani clade, and its minimum age may be much greater when the lignosus lineage is considered, distinguishing STORM from a collection of neutrally decaying viral remnants.

STORM asymmetry also extends to gene architecture. The phylogenetic relationships and tandem organization of these paralogs are consistent with retention of one integrated polyprotein followed by three tandem duplications restricted to the capsid region. Capsid-copy excess also occurs beyond *Scheffersomyces* in our genomic survey, including among the integrated totivirus-like elements sequenced in *Debaryomyces* by Taylor and Bruenn (2009). In STORM, the phylogenetic and genomic evidence links this excess to tandem duplication after endogenization and preservation over millions of years.

Comparisons with independently decaying copies provide additional evidence of selective maintenance. Intact STORM arrays retain open reading frames, transcriptional activity, predicted structures, and sites under purifying selection, whereas pseudogenized TLC copies and some neighboring co-transferred genes show frameshifts, stop codons, truncation, and structural deterioration. We found no intact partial arrays; every detected partial array was pseudogenized. Detectable pseudogenes in yeasts are generally evolutionarily young and often species-specific, consistent with the rapid erosion of unconstrained sequences (Lafontaine and Dujon 2010). Four-copy coding arrays are also uncommon in hemiascomycete yeasts (Despons et al. 2011), and gene-family evolution in Serinales is strongly biased toward contraction (Feng et al. 2025). Preservation of the differentiated *TLC1* to *TLC4* architecture in at least seven genomes, including after inferred interspecific transfers, therefore supports selective maintenance consistent with evolutionary domestication.

STORM challenges the expectation that integrated RNA viral genes will enter a slower evolutionary regime. STORM's estimated amino-acid substitution rate was approximately seven times the shared viral background rate in the main analyses. Faster STORM evolution persisted across both topologies and calibration schemes, although the estimated rate ratios depended on the assumed host-based ages.

*TLC4* illustrates how rapid evolution can coexist with selective preservation. Despite strong gene-wide relaxation, it retains a totivirus-like capsid architecture and constrained residues associated with cap binding and RNA decapping (Fig. 6; Table S8). These include homologs of L-A Tyr150, Asp152, catalytic His154, Tyr452, and Tyr/Phe538, which are separated in primary sequence but converge in the viral cap-binding pocket. These positions were either invariant or showed targeted evidence of purifying selection, whereas positive or directional selection was not detected at them. In contrast, *TLC1* to *TLC3* retain paralog-specific residue combinations at the homologous positions rather than the decapping-associated configuration.

The ancestral L-A virus system provides a mechanistic context for this asymmetry. Cap-snatching sites are active in both Gag and the Gag domain of Gag-Pol, and polymerase activity can activate cap snatching by a separate Gag molecule (Fujimura and Esteban 2019). STORM separates these ancestral features: *TLC2* retains the capsid-RdRp organization, whereas *TLC4* best preserves the decapping-associated configuration. This pattern is consistent with functional partitioning after tandem duplication. Because the initial decapping step can occur without viral transcription, *TLC4* could retain cap-binding or cap-removal activity without an RdRp domain. Full cap transfer, cooperation between *TLC2* and *TLC4*, and the biochemical functions of the paralogs remain hypotheses for experimental testing.

### STORM, RNA metabolism, and potential host benefits

Expression of a *D. hansenii* capsid paleogene can protect against a related virus (Warner et al. 2018). Because the *D. hansenii* capsid-like paleogenes were the closest yeast relatives of STORM detected here, this finding provides a relevant precedent for capsid-mediated interference. L-A virus supports killer satellites that can provide a competitive advantage, and recent work found that L-A itself increased host fitness under some environmental stresses (Hsiao et al. 2025). Excessive L-A replication, however, can cause protein aggregation and proteotoxic stress (Chau et al. 2023). Chromosomal capture could retain a beneficial viral activity while placing its dosage under host control, potentially reducing the costs of infection. The differentiated paralogs make this hypothesis testable: *TLC1*, with its conserved predicted capsid-like structure, is the leading candidate for capsid-mediated interference; *TLC4* for cap-related effects on RNA; and *TLC2* for RNA binding, residual polymerase activity, or cooperation with *TLC4*. *TLC3* remains functionally uncharacterized.

Expression analyses show that STORM is not a silent genomic relic. *TLC1* is consistently the most abundant array transcript across the sampled species and strains, and previously published mass spectrometry detected TLC1-derived peptides in *S. stipitis* (Taylor et al. 2013). *TLC2* to *TLC4* are expressed at lower levels and show distinct responses to growth conditions. These patterns are consistent with regulatory differentiation but do not identify the biochemical functions of the paralogs.

The growth-condition contrasts provide a possible physiological context for STORM expression, but they were not designed to test responses to viral infection. Baffled and shake cultures differ in oxygen transfer and turbulence, whereas chemostat and batch cultures differ in nutrient availability, growth rate, and metabolic state. STORM expression could therefore be connected to RNA metabolism, environmental physiology, or antiviral defense, but the present experiments cannot distinguish among these possibilities.

### Horizontal transfer, localized replacement, and loss of STORM

Our ASTRAL phylogenomic tree and the analysis of Opulente et al. (2024) recovered the same principal *Scheffersomyces* relationships. Against this consistent genomic background, the repeated STORM discordances and the localized replacement in *S. stipitis* NRRL Y-7124 support interspecific transfer of the locus. The shared insect-gut and rotten-wood habitats of these yeasts provide opportunities for genetic exchange, but ecological contact alone does not identify the transfer mechanism.

Gene concordance factors calculated from uncollapsed nuclear gene trees were high for the lignosus/shehatae clade (99.0%), the titani/stipitis clade (99.6%), and the grouping of the two *S. stipitis* strains (97.3%; Fig. 8), indicating that conflicts with these relationships were rare regardless of branch support. Among the 3,527 single-copy nuclear gene trees containing all 18 taxa, none recovered all relationships defining any of the three STORM discordances with strong support. Together with the local genomic evidence, this argues against incomplete lineage sorting as their explanation. Introgression remains possible, but explaining HGT1 and HGT2 this way would require at least two episodes of interspecific hybridization, followed by recombination and loss of donor ancestry outside the retained STORM-containing segments. Although the nuclear loci sample much of the coding genome, they show no evidence that the donor ancestry inferred for STORM extends widely across the genome. Small introgressed tracts remain possible.

**Figure 8:**
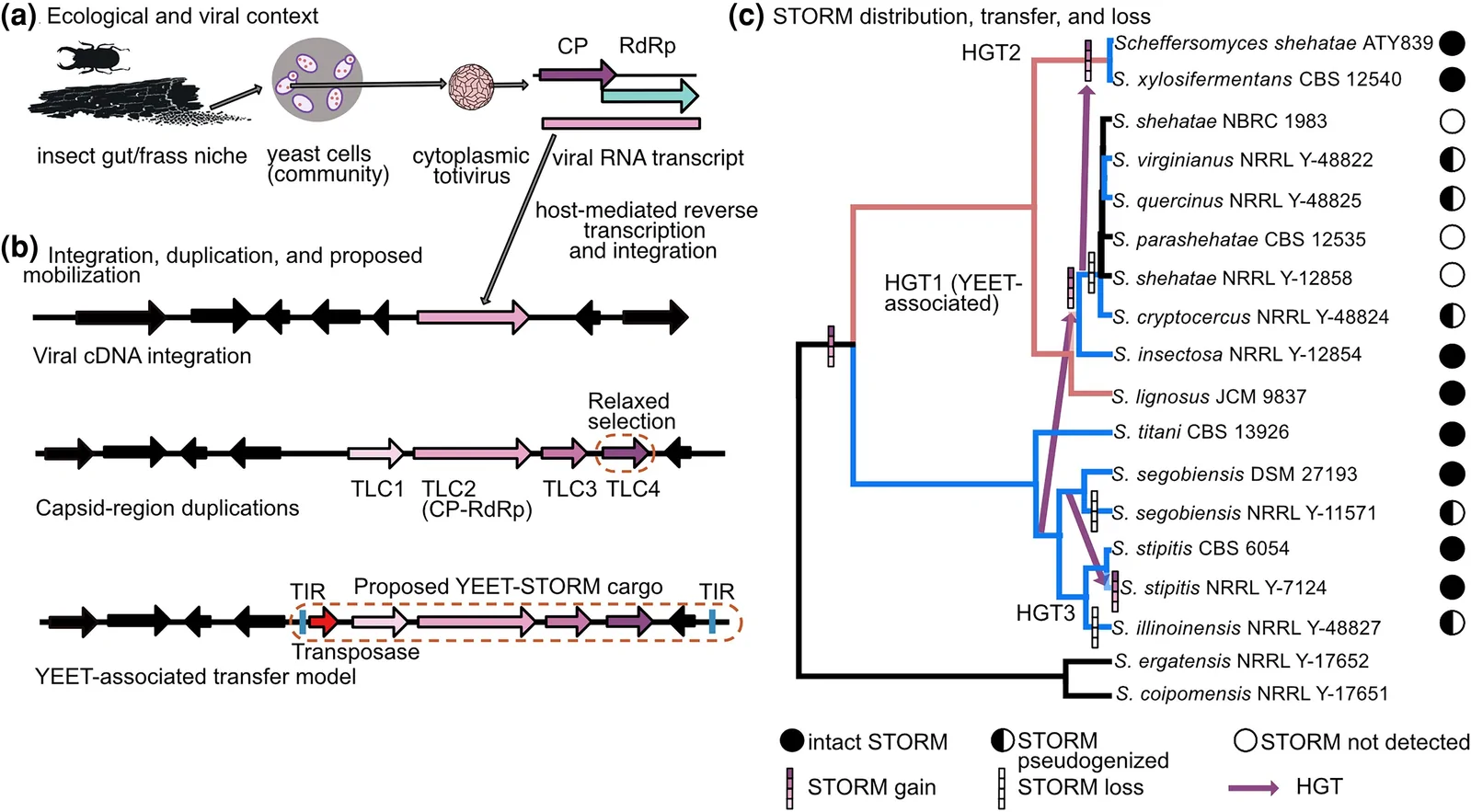
Proposed origin, asymmetric evolution, and horizontal transfer of the STORM paleoviral array. a) *Scheffersomyces* yeasts occupy insect-associated and rotten-wood/frass niches and harbor cytoplasmic totiviruses. Totiviral RNA encodes CP and RdRp, produced as a CP-RdRp fusion. b) Proposed integration and tandem duplication of an ancestral viral sequence, with *TLC2* retaining the CP-RdRp organization and *TLC1*, *TLC3*, and *TLC4* encoding capsid-like proteins. *TLC4* underwent relaxed selection while retaining predicted decapping-associated features. The dashed outline marks the proposed cargo for YEET-associated transfer. c) STORM distribution and inferred transfers mapped onto the ASTRAL species-tree cladogram estimated from 3,689 single-copy nuclear gene trees excluding STORM proteins. All internal branches had local posterior probability 1.0. Gene concordance factors were 99.0% and 99.6% for the lignosus/shehatae and titani/stipitis clades, and 97.3% for the two *S. stipitis* strains. No complete nuclear gene-tree match to any of the three STORM discordances was recovered in the supported-node analysis. Filled circles indicate intact STORM, half-filled circles indicate pseudogenized remnants, and open circles indicate no detected STORM. Arrows mark the three inferred HGT events. Red and blue branches indicate inferred STORM ancestry from the lignosus and titani clades, respectively; black branches indicate loss or absence.

For HGT1, several observations make YEET-associated transfer the most parsimonious explanation. YEET belongs to the Tc1/mariner superfamily, which is especially prone to horizontal transfer among fungi (Romeijn et al. 2026). The *S. insectosa* locus combines the complete STORM array with *ATP10* and YEET-transposase pseudogenes that group with titani-clade sequences, together with YEET1a-like TIRs. This TIR subtype is common in the *S. segobiensis/S. stipitis/S. titani* group and otherwise occurs in the lignosus/shehatae clade only among phylogenetically discordant YEET elements. Although *Scheffersomyces* genomes undergo frequent chromosomal rearrangements, rearrangement within the recipient genome cannot explain why STORM and its adjacent *ATP10*, YEET, and TIR sequences show affinities to the inferred donor *S. titani* clade. The pseudogenes could have been retained through linkage following either YEET-associated transfer or introgression, but the donor-associated TIR subtype and independent evidence of YEET mobility favor YEET-associated locus transfer. The complex TIR organization is consistent with sequential or nested YEET activity followed by erosion. The expected two-base-pair 5′-TA-3′ target-site duplication was not recovered, potentially because subsequent sequence changes obscured it. The cellular mechanism of HGT2 remains unresolved.

Expanded taxon sampling recovered a monophyletic STORM clade, and concordant discordances across multiple paralogs support movement of linked arrays in HGT1 and HGT2. In contrast, HGT3 replaced *TLC1*-*TLC3* in *S. stipitis* NRRL Y-7124 from a segobiensis-like donor, while *TLC4* and both flanks retained *S. stipitis* ancestry. Thus, complete arrays can move between species, while part of an array can subsequently be replaced without disrupting the four-copy architecture.

STORM has also been lost or reduced to pseudogenized fragments in several lineages, even where the surrounding genomic region remains conserved. A notable strain-level contrast occurs in *S. segobiensis*: the strain retaining intact STORM had no detected exogenous totivirus, whereas the strain without an intact array was the only strain in our survey that harbored one. The phylogenetic placement of this virus is consistent with host switching across a yeast genetic-code boundary (Taylor et al. 2013). This strain-level contrast raises the hypothesis that STORM influences totivirus persistence. Recurrent losses suggest that selection maintaining STORM depends on its genomic and ecological context.

### Paleoviral arrays as natural experiments in evolutionary rate and genome mobility

STORM reveals a second stage of mobility seldom considered after viral endogenization: an intact paleoviral array, already duplicated and differentiated, moved between congeneric yeast species (Fig. 8). At the ecological scale, STORM can be viewed as a complex genetic module of viral origin shared among species in rotten-wood and frass-associated yeast communities. Horizontal transfer of multigene regions is known in fungi, but, to our knowledge, no comparable case has been reported involving an intact tandem array derived from a nonretroviral RNA virus.

Host-calibrated rate analyses indicated that STORM evolved substantially faster than the sampled viral lineages, while selection analyses identified the strongest relaxation in *TLC4*. Endogenization placed STORM in a new selective context. Tandem duplication then appears to have enabled asymmetric evolution by allowing paralogs to retain different degrees of ancestral viral constraint, while selection preserved the four-copy architecture and specific structural features. Rapid divergence may eventually obscure their viral ancestry, causing ancient domestications to be missed by sequence-based searches. Mobile paleoviral arrays may therefore be an underrecognized source of genomic innovation in fungi.

## Materials and methods

### Data collection and identification of totivirus-like sequences

We compiled totivirus and totivirus-like sequences from the NCBI nonredundant (nr) protein database using BLASTP with yeast L-A capsid and RdRp proteins as queries (capsid: AAA50320.1; RdRp: AAA50321.1) and additional related viral sequences. Candidate exogenous totiviruses were retained if they contained (i) a recognizable totivirus-like capsid region and (ii) conserved RdRp motifs. We use the term “paleovirus” rather than “endogenous viral element” because “endogenous virus” has a distinct meaning in the totivirus literature, referring to persistent vertically transmitted dsRNA viruses (e.g. Fujimura and Wickner 1989; Schmidt et al. 2024; Hsiao et al. 2025). We use nonretroviral integrated RNA virus; Taylor and Bruenn 2009) synonymously in figure labels. To identify totivirus-like paleoviruses in yeast genomes, we screened publicly available assemblies from CTG-clade yeasts (Debaryomycetaceae; Serinales), in which the CUG codon is translated as serine rather than leucine, using TBLASTN with totivirus capsid and RdRp queries. We retained hits with *E*-value < 1 × 10^−5^ and alignment length ≥ 300 amino acids. Candidate loci were refined by ORF prediction (minimum ORF length 300 nt) under the appropriate yeast nuclear genetic code (i.e. genetic code 12 for Serinales).

### ORF calling and array annotation

Open reading frames were identified with NCBI ORFfinder using a minimum length of 300 nt, genetic code 12, and nested ORFs ignored. Capsid- and RdRp-like regions were identified by similarity to totivirus proteins and the presence of conserved domain-associated motifs. A polyprotein was defined as a continuous ORF containing both capsid- and RdRp-like regions. Within intact STORM arrays, *TLC1* to *TLC4* were assigned according to conserved array position and phylogenetic orthology across species. *TLC2* alone retains continuous capsid- and RdRp-like regions, whereas *TLC1* to *TLC4* contain only the capsid-like region. Nonoverlapping capsid- and RdRp-like loci were counted separately in each genome, and tandem copies were distinguished from independent loci using their genomic coordinates and flanking synteny. Because assembly completeness varies among species, genome-wide capsid and RdRp copy-number comparisons were treated as descriptive, particularly for loci outside intact STORM arrays. Annotated coding sequences were exported in FASTA format for downstream analyses.

### Microsynteny and macrosynteny analysis

To assess microsynteny around each distinct STORM context, we extracted every STORM-containing genomic region together with its flanking sequence, including the *S. titani* CBS 13926 region JAJMBD010000002.1:430000-519758, and searched each region against *Scheffersomyces* genome assemblies using BLASTN. Homologous regions from array-lacking genomes were also extracted, and flanking ORFs were predicted as described above.

Contiguous high-scoring blocks with a combined bit score ≥ 200 and *E* < 1 × 10^−5^ were used to delineate syntenic regions. Genomes with syntenic flanks but lacking array sequences were scored as array-lacking, whereas those with both flanks and internal capsid-like hits were scored as array-present. Putative flanking genes were annotated by ORF prediction and BLASTP searches against UniProt. Microsynteny maps were constructed by aligning homologous STORM-containing and array-lacking regions with MAFFT (Katoh and Standley 2013) and comparing gene order relative to the *Scheffersomyces* species phylogeny. Chromosome-scale synteny was assessed with ntSynt v1.03 (Coombe et al. 2025a) and ntSynt-viz v1.0 (Coombe et al. 2025b) using distance thresholds -d 2 and -d 20. Chromosome orientations were normalized relative to *S. shehatae* NBRC 1983 using the “normalize strand” option, and blocks containing STORM were examined for rearrangements and translocations.

### Time-calibrated species phylogeny

All focal species are represented in the calibrated tree of Opulente et al. (2024). To place additional strains (*S. segobiensis* DSM 27193 and *S. shehatae* JCM 18690 = ATY839), we compiled ten informative single-copy nuclear genes used in yeast phylogenetics (Aguileta et al. 2008; Taylor et al. 2013; Table S9), aligned them with MAFFT, and inferred a partitioned maximum-likelihood tree in IQ-TREE 3. Divergence times were estimated in MEGA 12 with RelTime using internal calibrations from TimeTree 5 (Kumar et al. 2022), including the estimated *S. stipitis–S. lignosus* divergence of 54 Ma, with 43.2 and 65.1 Ma used as bounds (Shen et al. 2018, 2020).

### Viral and paleoviral phylogeny

For viral and paleoviral phylogenies, we compiled amino-acid sequences of capsid domains from exogenous totiviruses, singleton totivirus-like paleoviruses, and STORM copies (*TLC1* to *TLC4*) from all sampled species. Sequences were aligned using MAFFT v7 (E-INS-i, with G-INS-i for larger datasets). To assess the effect of alignment on the results, we used ClipKIT 2.1.3 (kpic-smart-gap mode; Steenwyk et al. 2020). Sequence metadata for the 84-taxon capsid amino-acid alignment underlying the main capsid protein (CP) tree (host species, strain or isolate, primary accession, and re-parsed genomic coordinates) are provided in Data S2. The underlying alignment (84 sequences × 956 amino-acid columns) is provided as Data S3.

Maximum-likelihood trees were inferred in IQ-TREE 3 using ModelFinder (Kalyaanamoorthy et al. 2017; Wong et al. 2026). Branch support was assessed with SH-aLRT and 1,000 ultrafast-bootstrap replicates. The nt-based phylogeny in Fig. 3 was restricted to intact STORM ORFs in the ingroup, with Tuber aestivum virus 1 and *L. beijingensis* virus L-A used as outgroups. The length-variable C-terminal region corresponding to approximately 60 amino acids was excluded before analysis. MixtureFinder (Ren et al. 2025) selected a six-component nucleotide substitution mixture model with invariant sites and four gamma rate categories for the 34-sequence nucleotide alignment (Data S4). To assess sensitivity to heterotachy, we also fitted GHOST models with two, three, or four classes (Crotty et al. 2020), each from two independent starts. We compared model fit using the Bayesian information criterion (BIC) and assessed the stability of the inferred relationships. RdRp domains were aligned separately; the 43-sequence, 737-column alignment underlying Fig. 5c and Figure S2 is provided as Data S5.

The full capsid and RdRp trees were rooted with the related plant virus black raspberry virus F (EU082131.1), whereas the STORM codon tree was rooted with Tuber aestivum virus 1 and *L. beijingensis* virus L-A. Trees were visualized in iTOL (Letunic and Bork 2024), and root-to-tip patristic distances were computed with Biopython.

### Tests of horizontal transfer of paleoviral arrays among yeast genomes

To quantify the genome-wide background of gene-tree discordance, we removed all TLC/STORM proteins from the predicted proteomes of 18 taxa before orthogroup inference; *S. coipomoensis* and *S. ergatensis* were included as outgroups. Strain-matched proteins were obtained from available annotations or generated from genomic coding sequences using the Alternative Yeast Nuclear code (NCBI Table 12) where appropriate. OrthoFinder v3.1.5 (Emms et al. 2026) identified single-copy orthogroups, which were filtered for taxon representation, alignment length, and phylogenetic informativeness. MAFFT alignments and independently inferred IQ-TREE 3 gene trees yielded 3,689 primary loci and a strict sensitivity set of 3,527 loci containing all 18 taxa.

Species trees were estimated with ASTRAL-IV v1.23.4.6 as implemented in ASTER (Zhang et al. 2025) from primary and strict gene-tree sets after collapsing edges below ultrafast-bootstrap thresholds of 10%, 50%, and 70%, and from uncollapsed trees. HGT-specific node tests were then applied to the 3,527 complete 18-taxon gene trees. A node was counted only when the corresponding original IQ-TREE edge had SH-aLRT ≥ 80 and ultrafast bootstrap ≥ 95. HGT1 required the *S. insectosa*/remnant-bearing recipient lineage to join the *S. segobiensis*/*S. stipitis*/*S. illinoinensis* group before *S. titani*. HGT2 required both the placement of the *S. xylosifermentans*/*S. shehatae* ATY 839 pair with the *S. cryptocercus*/*S. quercinus*/*S. parashehatae*-side group and the encompassing *S. insectosa* relationship. HGT3 required *S. stipitis* NRRL Y-7124 to group with *S. segobiensis* while the titani clade remained intact. Complete matches also required a strongly supported *S. ergatensis*/*S. coipomoensis* outgroup; HGT2 additionally required support for the *S. xylosifermentans*/*S. shehatae* ATY 839 pair itself. Trees lacking an HGT-defining node or its required support were classified as partial or weak/other. These topology tests do not determine transfer direction. To test whether the discordant relationships were supported independently by each paralog in STORM, we evaluated separate *TLC1* to *TLC4* amino-acid alignments against two candidate topologies: the maximum-likelihood STORM topology and the corresponding species-tree-constrained topology. Topologies were compared using approximately unbiased tests with 10,000 replicates of the resampling estimated log-likelihoods (RELL) procedure (Shimodaira 2002).

To test the ancestry of host-derived sequences associated with the *S. insectosa* STORM locus, we analyzed nucleotide alignments containing the STORM-linked *ATP10* and *SKI3* pseudogene fragments together with intact orthologs from representative *Scheffersomyces* species. Maximum-likelihood trees were inferred with IQ-TREE 3 using ModelFinder and rooted with *S. coipomoensis*. The *ATP10* alignment contained 20 sequences and 844 columns and was analyzed under TN + F + G4. The *SKI3* alignment contained 19 sequences and 4,224 columns and was analyzed under TIM2 + F + I + G4. Branch support was assessed with 1,000 SH-aLRT replicates and 1,000 ultrafast-bootstrap replicates, with bootstrap nearest-neighbor interchange optimization. The paired trees are shown in Figure S4.

As an earlier targeted comparison, we assembled 272 translated reference loci for *S. stipitis*, *S. lignosus*, and *S. xylosifermentans*, with *L. beijingensis* as the outgroup. Alignments were excluded when the outgroup sequence was shorter than 500 nt, ingroup alignments contained more than 50 indel gaps, or significant ingroup base-composition differences were detected. Patristic-distance summaries are provided in Data S8, with alignments and IQ-TREE outputs on Zenodo. This targeted survey was treated as descriptive support; the 18-taxon analysis provided the primary genome-wide evaluation of discordance.

To assess movement of YEET, a Tc1/mariner/IS630-superfamily transposase family, we constructed a phylogeny of YEET-like sequences in *Scheffersomyces*. The full-length *S. shehatae* protein BDMP01000001.1:2358241-2359608 was used as a query in TBLASTN searches of *Scheffersomyces* whole-genome shotgun assemblies; the distantly related *S. spartinae* was excluded. Fragmented pseudogenes were reconstructed with TFASTY using the Müller–Vingron VT120 matrix. Hits were parsed into FASTA format, aligned with MAFFT, and filtered with ClipKIT in smart-gap mode, which removed 34% of sites. Sequences shorter than 150 amino acids were excluded. We inferred a maximum-likelihood phylogeny in IQ-TREE 3, selected the substitution model with ModelFinder, and assessed support with SH-aLRT. The final alignment comprised 213 YEET transposases plus the *Circinella minor* IS630 outgroup (214 sequences × 950 columns; Data S6). TIRs were identified with BLASTN using diagnostic 13-bp core consensus seeds (15 bp for mYEET2b), and their boundaries were refined with sliding-window inverted-repeat alignments. For the nonautonomous mYEET2b MITE, TIR length was defined as the genome-BLAST-confirmed 40-bp span of the bracketing repeats. Confirmed elements had a median aligned TIR length of 191 bp (range, 15 to 201 bp). Data S1 provides element coordinates, TIR orientations, subfamily assignments, and transposase-clade membership; Table S2 summarizes counts and median TIR and element lengths by subfamily and clade.

### Genetic distance and rate comparisons

To visualize accumulated divergence among predefined groups, pairwise and root-to-tip patristic distances were compared with a two-sided Mann–Whitney *U* test and Benjamini–Hochberg correction. These are descriptive distance summaries rather than formal tests of evolutionary rate. Six groups were analyzed: exogenous totiviruses, non-STORM paleoviruses, and *TLC1* to *TLC4*. Distances were calculated from maximum-likelihood trees with Biopython; statistical analyses used NumPy and SciPy, and figures used Matplotlib. Formal amino-acid rate comparisons used PAML local-clock models described below. OpenAI tools assisted development and debugging of custom scripts; the authors verified all inputs, code, and outputs.

### Local-clock tests of amino acid substitution rates

The primary local-clock analysis used 46 capsid amino-acid sequences, excluding pseudogenized *TLC4* copies from *S. illinoinensis* NRRL Y-48827 and *S. segobiensis* NRRL Y-11571 and singleton non-STORM paleoviruses. To assess sensitivity to sparse paleoviral sampling, these sequences were aligned with 17 additional capsid-like paleoviral ORFs remaining after the removal of three near-duplicates from 20 candidates. Candidates were 600 to 750 amino acids long, contained no stop codons, and covered ≥85% of the conserved fungal-virus capsid core. The added sequences were then removed to produce the 46-sequence, 887-position alignment used for rate estimation. We evaluated two fixed topologies using the same calibration scheme. The main topology placed STORM with the *L. beijingensis* virus/Tuber aestivum virus 1 lineage, consistent with the full capsid phylogeny (Fig. 2) and the RdRp phylogeny. The alternative topology, recovered from the reduced-taxon capsid analysis, placed STORM outside the sampled exogenous fungal-virus clade. Both topologies retained STORM monophyly. Substitution models were evaluated with ModelFinder in IQ-TREE v3.1.3 on the same alignment under each fixed topology. Q.YEAST+Γ4 had the lowest BIC among the tested models using four gamma rate categories under both topologies; the broader model search preferred Q.YEAST + R4. We used Q.YEAST+Γ4 (Minh et al. 2021) for calibrated local-clock analyses in PAML v4.10.10/codeml (Yang 2007), importing the Q.YEAST substitution matrix and using its amino-acid frequencies. The gamma shape parameter was estimated.

Time was scaled so that one PAML unit equaled 100 Myr. The STORM crown was fixed at 54 Ma, with 43.2 and 65.1 Ma used as sensitivity bounds. Each STORM age was evaluated with the node joining *Torulaspora delbrueckii* virus and the six sampled *Saccharomyces* L-A viruses fixed at 97 Ma, based on the corresponding host divergence (Kumar et al. 2022). In an alternative calibration scheme, we replaced the 97-Ma constraint with a host-based age assigned to the MRCA of *L. beijingensis* virus and the sampled *Saccharomyces* L-A virus lineage. The corresponding host divergence, represented by *Lodderomyces elongisporus* and *S. cerevisiae* in the dated phylogeny of Shen et al. (2018), had a mean age of 235.1 Ma and a 95% credible interval of 198.5 to 277.6 Ma. We paired each of the three STORM ages with the mean and both bounds of the deeper host calibration, giving nine age combinations. We fitted H0 and H1 to each combination under both topologies. Under the deeper calibration scheme, the model estimated the age of the Torulaspora-Saccharomyces viral node rather than fixing it at 97 Ma.

The null model (H0) assigned one substitution rate to all branches. The alternative model (H1) assigned one rate to branches within the STORM crown, and another to all remaining branches, including the incoming STORM stem and Black raspberry virus F. Each H0 to H1 comparison used the same fixed topology and calibrations; the models differed by one rate parameter and were compared using a likelihood-ratio test with one degree of freedom.

Each model was fitted from at least two starting conditions, with additional runs used to resolve convergence warnings or differences in likelihood. We retained the highest-likelihood fit after confirming agreement between independent starts. As a substitution-model sensitivity analysis, we repeated the comparisons under JTT + F+Γ4 using the same alignment, topologies, calibrations, and rate assignments (Table S6).

Both calibration schemes assumed that the selected viral divergences coincided with the corresponding host divergences.

### Tests for selection and relaxation

We tested for shifts in selection intensity with RELAX (Wertheim et al. 2015), where *K* < 1 indicates relaxation and *K* > 1 indicates intensification. Analyses used HyPhy with the Alternative Yeast Nuclear code (NCBI Table 12), a codon general time-reversible (GTR) model, and three ω classes. The 34-sequence STORM alignment and fixed phylogeny were used for contrasts of all STORM branches vs. viral capsids, *TLC4* vs. other *TLCs*, and *TLC1* vs. *TLC2*/*TLC3*. Analogous analyses were applied to *SKI3, SKI2, and DCP2*. To test whether *TLC4* relaxation concealed episodic diversifying selection, BUSTED analysis version 4.6 (Murrell et al. 2015) was run in HyPhy 2.5.84 on the 648-codon alignment of eight intact *TLC4* sequences and the corresponding pruned phylogeny. All *TLC4* branches were assigned to the test set, synonymous-rate variation was enabled, three ω classes were fitted, and the Alternative Yeast Nuclear genetic code was used.

To identify codons with different nonsynonymous substitution rates among capsid paralogs, we applied Contrast-FEL (Kosakovsky Pond et al. 2021) as implemented in HyPhy (analysis version 0.61). We used the same in-frame codon alignment as for RELAX (34 taxa, 658 codons; Alternative Yeast Nuclear genetic code) and the corresponding maximum-likelihood phylogeny. Branches leading to each tandem capsid paralog were labeled according to array membership (*TLC1*, *TLC2*/3, or *TLC4*), with viral outgroups and remaining branches treated as background. Contrast-FEL jointly estimates site-specific synonymous (α) and nonsynonymous (β) substitution rates for predefined branch sets and tests whether β differs among groups using likelihood-ratio tests with permutation-based *P*-values and alignment-wide FDR correction. We conducted three pairwise contrasts (*TLC1* vs. *TLC4*, *TLC2*/3 vs. *TLC4*, and *TLC1* vs. *TLC2*/3), enabling synonymous-rate variation and permutation-based inference. A nominal *P*-value threshold of 0.05 and an FDR *q*-value threshold of 0.20 were used to identify codons with significantly different nonsynonymous rates between branch sets.

In a separate positional analysis corresponding to Figure S6, the eight terminal *TLC4* branches were designated as foreground and the remaining 57 branches as background. This Contrast-FEL analysis tested for sites with different nonsynonymous rates between *TLC4* and the remaining phylogeny using alignment-wide FDR correction; *q* ≤ 0.20 was used for this exploratory sitewise comparison.

To assess site-specific selection, we ran separate FEL analyses in HyPhy 2.5.84 (FEL analysis version 2.6; Kosakovsky Pond and Frost 2005) on two datasets: (1) eight intact *TLC4* sequences in a 648-codon *TLC4*-only alignment, and (2) 24 *TLC1*/2/3 sequences in the 658-codon S4 alignment. Before the *TLC4* analysis, the two viral sequences were removed from the codon alignment and pruned from the corresponding maximum-likelihood tree. The *TLC1*/2/3 phylogeny was pruned from the full S4 topology. All branches in each tree were tested, and synonymous-rate variation was enabled (the Alternative Yeast Nuclear genetic code was used). Positions were mapped to the S4 coordinate system. Seven decapping-associated positions (S4 positions 153 to 157, 460, and 564) were specified a priori from structural comparisons and the YHDGH, YDDGH, and YNDGH forms observed among sampled extant totiviruses.

We applied two multiple-testing corrections to address different questions. To test whether constraint persisted anywhere across *TLC4* despite gene-wide relaxation, raw FEL *P*-values were corrected by the Benjamini–Hochberg procedure across all 648 codons; *q* ≤ 0.10 was used to identify supported sites, with *q* ≤ 0.05 also reported. To test the prespecified decapping hypothesis, BH correction was applied separately to the relevant positions in each dataset. Two *TLC4* positions were invariant and therefore uninformative for FEL, leaving five testable *TLC4* positions; all seven homologous *TLC1*/2/3 positions were testable. Targeted *q* ≤ 0.10 was treated as evidence of localized constraint, with *q* ≤ 0.05 noted as stronger support. Invariant positions were reported descriptively as conserved but were not included in the *TLC4* targeted correction.

To test directional amino-acid evolution on *TLC4* branches, we applied FADE as implemented in HyPhy 2.5 (Kosakovsky Pond et al. 2020) to the full S4 protein alignment, designating *TLC4* branches as foreground. FADE estimates empirical BFs for substitution bias toward each amino acid at each site; BF > 100 was treated as strong evidence.

To evaluate recombination as a potential confounder of the *TLC4* selection analyses, we applied GARD (Kosakovsky Pond et al. 2006) to the 1,944-nt, 648-codon alignment of eight intact *TLC4* sequences under the Alternative Yeast Nuclear code. GARD used a GTR model with among-site rate variation, compared single-tree and segmented models by AICc, and evaluated candidate breakpoint support sitewise.

### Structural prediction and comparison

Capsid-domain structures were predicted with AlphaFold3 (Abramson et al. 2024) for 80 viral and paleoviral sequences representing seven structural groups. Five seeds were used per job, and the highest-ranked seed was retained; all models are provided in Data S7. Per-residue pLDDT was extracted from the ModelCIF B-factor field. Seventy-nine structures had at least 70% of residues above pLDDT 70; the sole exception was a 170-residue singleton fragment (67.1%). In every structural group, the mean proportion of residues above 70 exceeded 84%. Group distributions are shown in Figure S7, and PAE heatmaps are provided in Figures S8 and S9. Foldseek comparisons (van Kempen et al. 2024) used exogenous viral structures as queries and query-normalized TM-score (qTM-score) as the primary similarity metric. To compare STORM proteins directly with an experimentally determined viral capsid, AlphaFold3 models of *TLC1* to *TLC4* from *S. stipitis* CBS 6054 were aligned independently to a capsid from the cryo-electron microscopy structure of *S. cerevisiae* L-A virus (PDB 8A5T, chain A; Schmidt et al. 2024) using Foldseek in TM-align mode. Foldseek TM-scores were recorded for each comparison. The overlays were visualized in the same orientation using UCSF ChimeraX.

Database creation and searching followed standard Foldseek protocols. Each paleoviral structure was compared against all exogenous viral references using a permissive *E*-value threshold (*E* ≤ 10,000) to ensure comprehensive coverage and assigned the maximum qTM-score obtained against any reference structure. Structures with no returned alignment were assigned a qTM-score of 0.0 to preserve the signal of complete structural divergence.

After initial identification, we retained singleton paleoviral structures only when the best Foldseek alignment covered at least 80% of the exogenous viral reference sequence (the query; *qcov* ≥ 0.80). This criterion reduced the singleton dataset from 32 to 17 structures. Statistical comparisons of qTM-score distributions used Kruskal–Wallis tests across all groups, followed by pairwise Mann–Whitney *U* tests for specific hypotheses of interest. All statistical analyses were performed in Python using SciPy v1.16.1.

We mapped evolutionary rate variation and selection metrics onto the predicted structures to visualize regions of relaxed constraint and enhanced divergence. Particular attention was given to motifs implicated in decapping and RNA binding based on experimentally characterized totivirus structures (Okamoto et al. 2020; Grybchuk et al. 2022).

### RNA-seq analysis

Paired-end RNA-seq data were obtained from NCBI SRA and JGI MycoCosm (Table S10). Reads were quality-trimmed and quantified with Salmon v1.10.3 against the corresponding full transcriptome augmented with *TLC1* to *TLC4* where required. Count matrices were imported with tximport using lengthScaledTPM and analyzed with DESeq2 size-factor normalization and Benjamini–Hochberg correction. Differential-expression inference was restricted to replicated contrasts: baffle vs. shake for *S. xylosifermentans* (four biological replicates per condition) and chemostat vs. batch for *S. stipitis* CBS 6054 (two per condition). FDR < 0.05 was treated as statistical support, whereas |log_2_FC| ≥ 1 was additionally required to meet the 2-fold effect-size criterion. FDR-supported changes below this threshold were described as statistically detectable but smaller than 2-fold. Single-sample datasets were used only for descriptive transcription estimates. Raw counts, normalized counts, complete DESeq2 outputs, metadata, scripts, validation, and session information are provided in Data S9.

For descriptive cross-sample plots of *TLC1* to *TLC4*, transcripts per million (TPM) values were scaled to the geometric mean of five candidate reference genes selected for low responsiveness in the replicated contrasts: *ACT1*, *DCP2*, *TFC1*, *SCW4*, and *SKI3*. The geometric mean used genes with TPM > 0, and samples with fewer than three detected reference genes were excluded. Candidate-gene stability is shown in Figure S12. Relative expression was calculated as TPM(*TLC*)/geometric mean [TPM(*ACT1*), TPM(*DCP2*), TPM(*TFC1*), TPM(*SCW4*), TPM(*SKI3*)].

Relative expression was multiplied by 100 and summarized by TLC and condition as the mean ± SE across biological replicates (Fig. 7). DESeq2 fold changes and FDR values, rather than reference-gene scaling, were used for inferential condition contrasts.

## Supplementary Material

msag269_Supplementary_Data

## Data Availability

Genome assemblies and STORM-locus coordinates are described in the Methods and Table S1; sequence metadata and accessions for the capsid analyses are provided in Data S2. Public RNA-seq accessions and sample metadata are provided in Table S10. Data S1 to S10 are deposited at Zenodo (https://doi.org/10.5281/zenodo.22900571). A portion of these data was produced by the US Department of Energy Joint Genome Institute (https://ror.org/04xm1d337; Contract No. DE-AC02-05CH11231).
