# Supplementary material for: A domesticated totivirus-like tandem array undergoes interspecific transfer and asymmetric evolution
Source: Mol Biol Evol. 2026 Oct 31;43(11):msag269. doi: 10.1093/molbev/msag269 (PMC13756089; doi:10.1093/molbev/msag269)
Supplement: Supplementary file 1 [file msag269_supplementary_data.pdf]

Supplementary Materials for:

A domesticated totivirus-like tandem array undergoes interspecific transfer and asymmetric evolution

Authors:

Derek J. Taylor[1]* and Dominic A. Tringali[1]

[1] Department of Biological Sciences, University at Buffalo, The State University of New York, Buffalo, NY, USA

*Corresponding author: djtaylor@buffalo.edu

This file includes Supplementary Figures S1-S12 and Supplementary Tables S1-S10. Ten supplementary data files accompany the article: S1, YEET element data; S2, metadata for the 84-taxon capsid alignment; S3, capsid amino acid alignment; S4, *Scheffersomyces* TLC nucleotide alignment; S5, RdRp amino acid alignment; S6, YEET transposase alignment; S7, AlphaFold3 structures and Foldseek/pLDDT/PAE outputs; S8, phylogenetic concordance summaries; S9, complete expression analysis files; and S10, expanded OrthoFinder/IQ-TREE/ASTRAL and HGT-specific topology outputs. Large files and outputs are archived at Zenodo (doi:10.5281/zenodo.22900571).

Supplementary Figures

Supplementary Figure S1. BLAST evidence for sequence-level conservation and lineage-specific distribution of the STORM region.

BLAST hits against the *S. shehatae* ATY839 STORM-containing contig (BDMO01000001.1) ranked by maximum bit-score. Left: BLAST summary. Right: graphical alignment of the same hits across coordinates ~1.78-1.81 Mb of the query contig. Red-shaded entries are the *shehatae*-clade *Scheffersomyces* species; blue-shaded hits are the *stipitis*-clade species, which carry STORM at a different chromosomal locus and therefore yield only short flanking-gene hits at this position.

Description	Max Score	Total Score	Query Cover	E value	Per. ident	Acc. Len	Accession
Scheffersomyces shehatae DNA, contig: ATY839_sc01a, strain: ATY839	71916	72599	100%	0.0	100.00	3488706	BDMO01000001.1
Scheffersomyces xylosifermentans strain CBS 12540 RJT20scaffold_4	45998	71114	100%	0.0	99.25	1826639	JAXBLS010000004.1
Scheffersomyces lignosus DNA, scaffold: scaffold_4, strain: JCM 9837	5550	16873	42%	0.0	83.58	1730773	BCGS01000005.1
Scheffersomyces shehatae DNA, contig: NBRC1983_sc05a, strain: NBRC 1983	5387	20197	49%	0.0	83.38	1741095	BDMP01000005.1
Scheffersomyces quercinus strain NRRL Y-48825 NODE_6_length_736209_cov_22.039_ID_4043	5378	20144	47%	0.0	83.39	736209	JAKTUQ010000006.1
Scheffersomyces cryptocercus strain NRRL Y-48824 NODE_29_length_209786_cov_64.2939_ID_6609	5377	19804	49%	0.0	83.34	209721	JANIVO010000029.1
Scheffersomyces virginianus strain NRRL Y-48822 NODE_32_length_168694_cov_29.7648_ID_6328	5371	19633	49%	0.0	83.33	168694	JAJMAT010000032.1
Scheffersomyces parashehatae strain CBS 12535 NODE_12_length_327702_cov_20.4121_ID_7521	5369	20141	49%	0.0	83.31	327702	JAJLYY010000012.1
Scheffersomyces insectosa strain NRRL Y-12854 NODE_35_length_122011_cov_17.2874_ID_12973	5314	20358	51%	0.0	83.04	122011	JAJMAO010000035.1
Scheffersomyces shehatae strain NRRL Y-12858 NODE_11_length_229527_cov_18.642_ID_7340	5310	11281	29%	0.0	83.07	229527	JAKTZA010000011.1
Scheffersomyces stipitis CBS 6054 tig00000005_pilon	1565	3806	14%	0.0	81.42	1591760	PQNB01000002.1
Scheffersomyces titani strain CBS 13926 NODE_1_length_606302_cov_33.5475_ID_7728	1565	4067	21%	0.0	81.42	606302	JAJMBD010000001.1
Scheffersomyces stipitis strain NRRL Y-7124 chromosome 3	1561	3799	14%	0.0	81.36	1880751	JADGGA010000004.1
Scheffersomyces illinoinensis strain NRRL Y-48827 NODE_7_length_476829_cov_15.7456_ID_7003	1549	3776	15%	0.0	81.05	476829	JAJMAP010000007.1

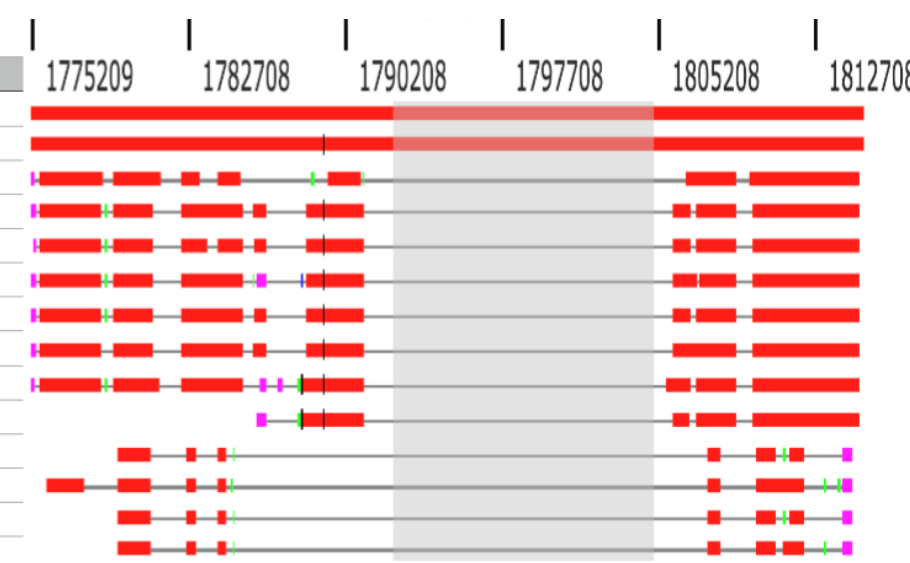

Supplementary Figure S2. Maximum-likelihood phylogeny of totivirus-like RNA-dependent RNA polymerases.

The Maximum likelihood estimate was inferred from amino-acid sequences and a Q.YEAST+I+G4 substitution model and was rooted with Black raspberry virus F. Red shading indicates STORM sequences associated with TLC2. Node labels show SH-aLRT/ultrafast-bootstrap support from 1,000 replicates.

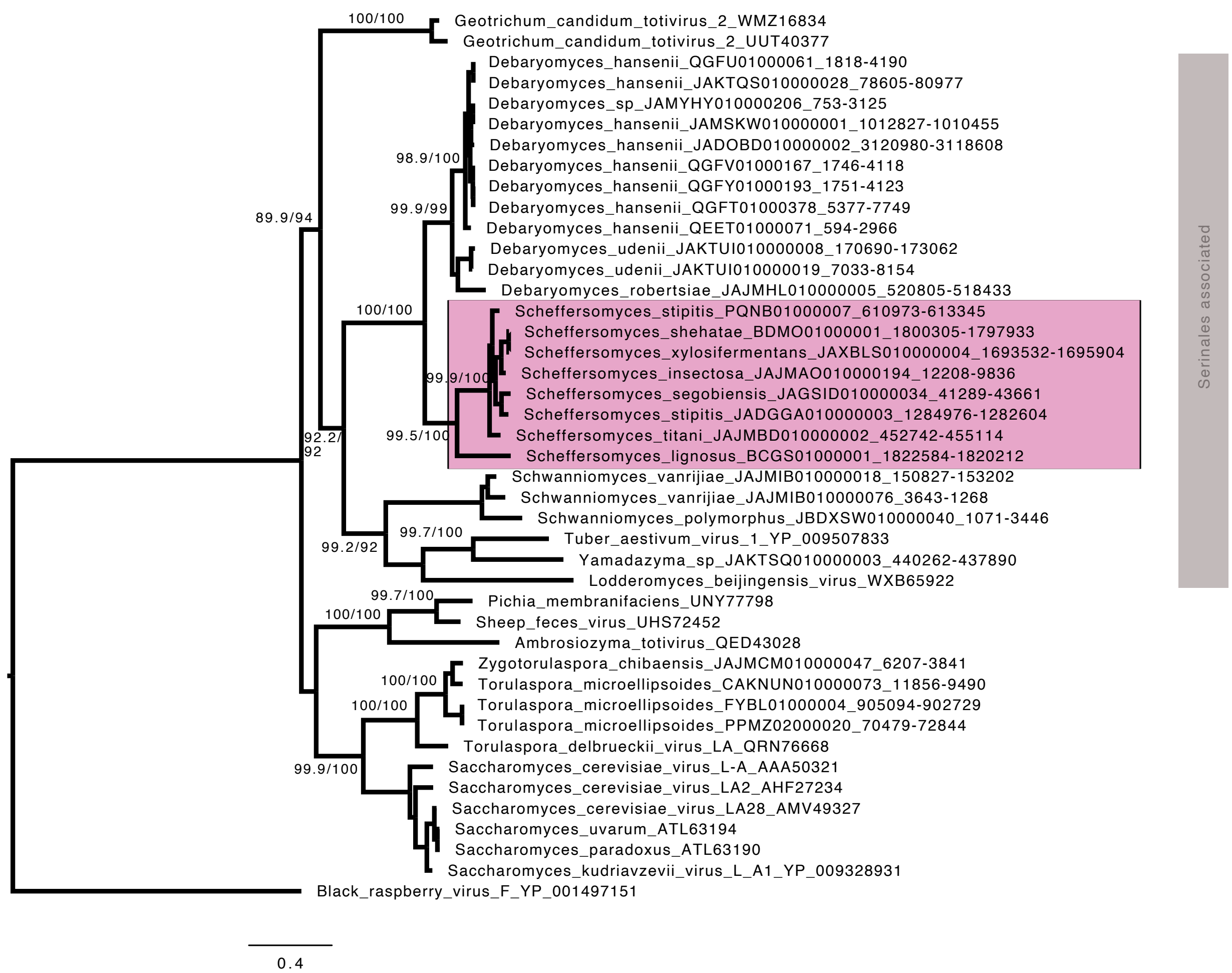

Supplementary Figure S3. Phylogeny and structural diversification of YEET transposases in *Scheffersomyces*.

Maximum-likelihood phylogeny of YEET-family transposases identified across genomes of *Scheffersomyces*, rooted with an IS630-family transposase from *Circinella minor*. Clades of transposase (YEET-α and YEET-β) are indicated by branch labels. Tip-associated colour strips denote terminal inverted repeat (TIR) subtype (YEET1a, YEET1b, or other), inferred from flanking sequence comparisons. Filled circles mark strong TIR support, whereas filled nodes indicate branches with strong approximate likelihood-ratio test (SH-aLRT) support. Dashed connectors identify putative recent horizontal transfers. Scale bar indicates substitutions per site. Transposase coordinates, TIR sequences, and subfamily/clade assignments are presented in Supplementary Data S1.

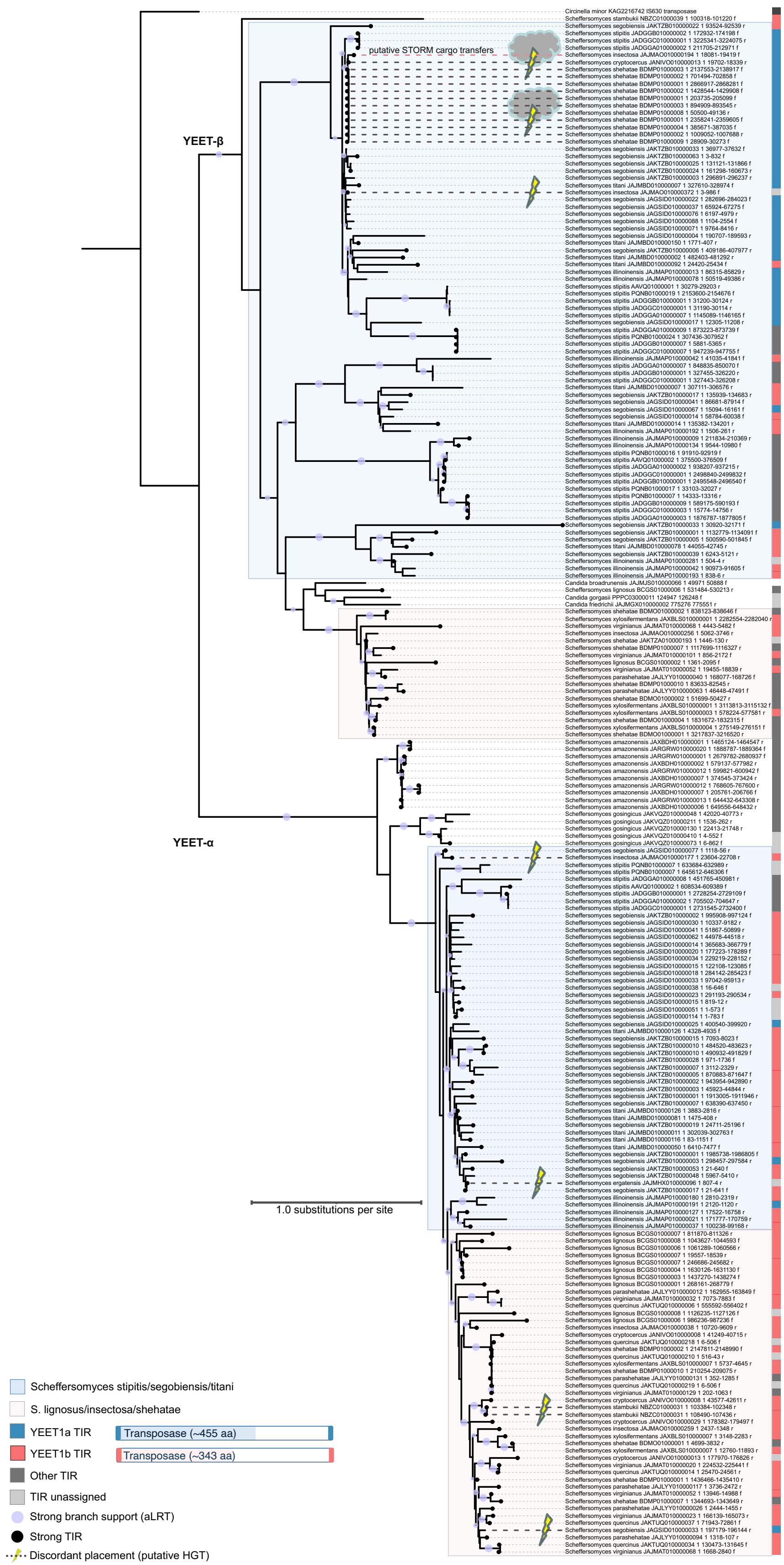
YEET-β
YEET-α
putative STORM cargo transfers
1.0 substitutions per site
Scheffersomyces stipitis/segobiensis/titani
S. lignosus/insectosa/shehatae
YEET1a TIR
Transposase (~455 aa)
YEET1b TIR
Transposase (~343 aa)
Other TIR
TIR unassigned
Strong branch support (aLRT)
Strong TIR
Discordant placement (putative HGT)

Supplementary Figure S4. Phylogenetic affinities of the *SKI3* and *ATP10* sequences associated with the *Scheffersomyces insectosa* STORM locus. Maximum-likelihood phylogenies with SH-aLRT/ultrafast-bootstrap percentages. Shaded rectangles indicate the two major clades of *Scheffersomyces* examined here. (A) *SKI3* (B) *ATP10 S. insectosa* sequences are highlighted in red, and trees were rooted using *S. coipomoensis*. Scale bars indicate nucleotide substitutions per site.

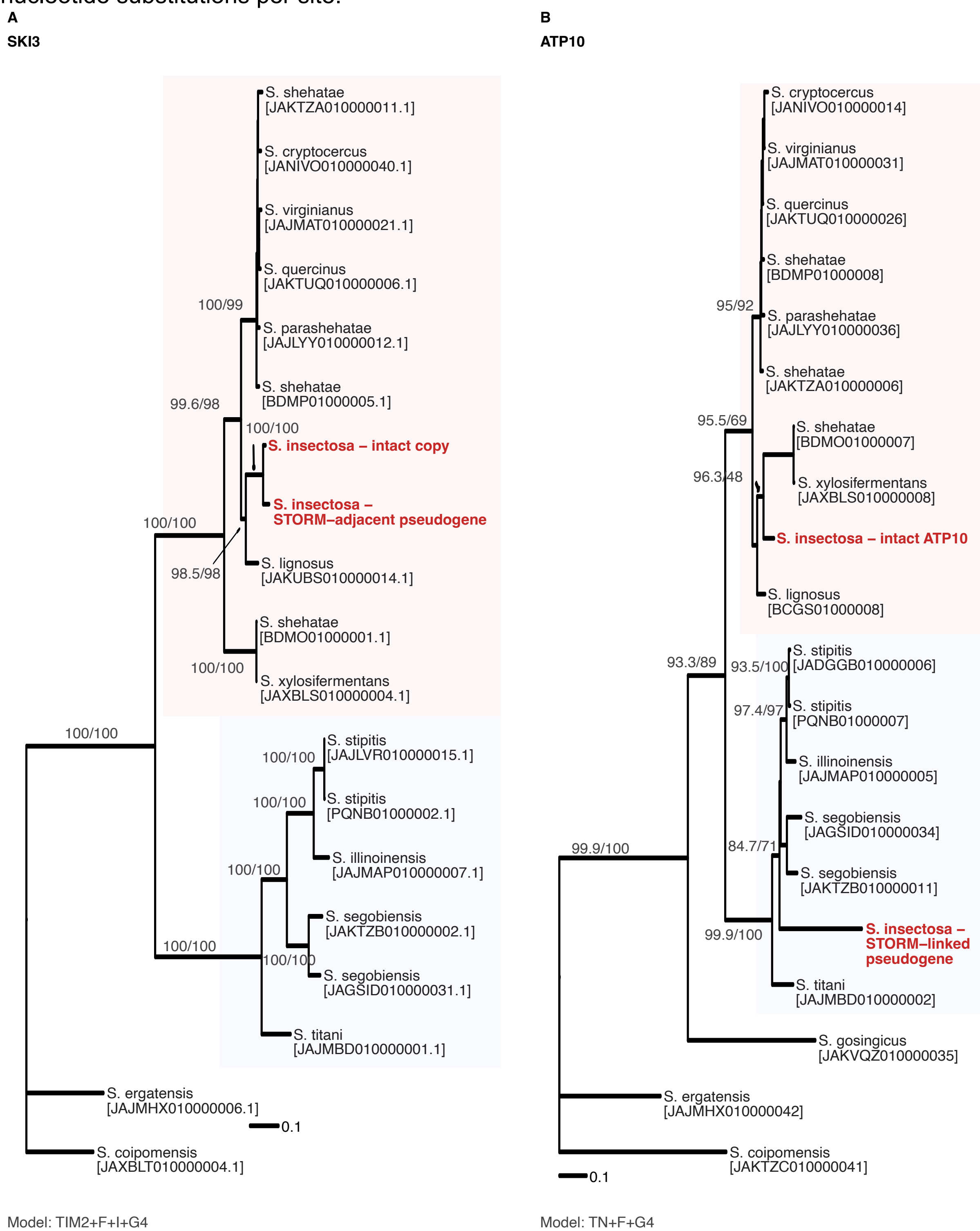

Supplementary Figure S5. Root-to-tip capsid (CP) distances after ClipKIT alignment trimming.

Box-and-strip plot of root-to-tip patristic distances (amino-acid substitutions per site) for the six groups plotted in Figure 5A after filtering the MAFFT capsid alignment with ClipKIT kpic-smart-gap (25.4% of sites removed). These values describe accumulated divergence rather than clock rate.

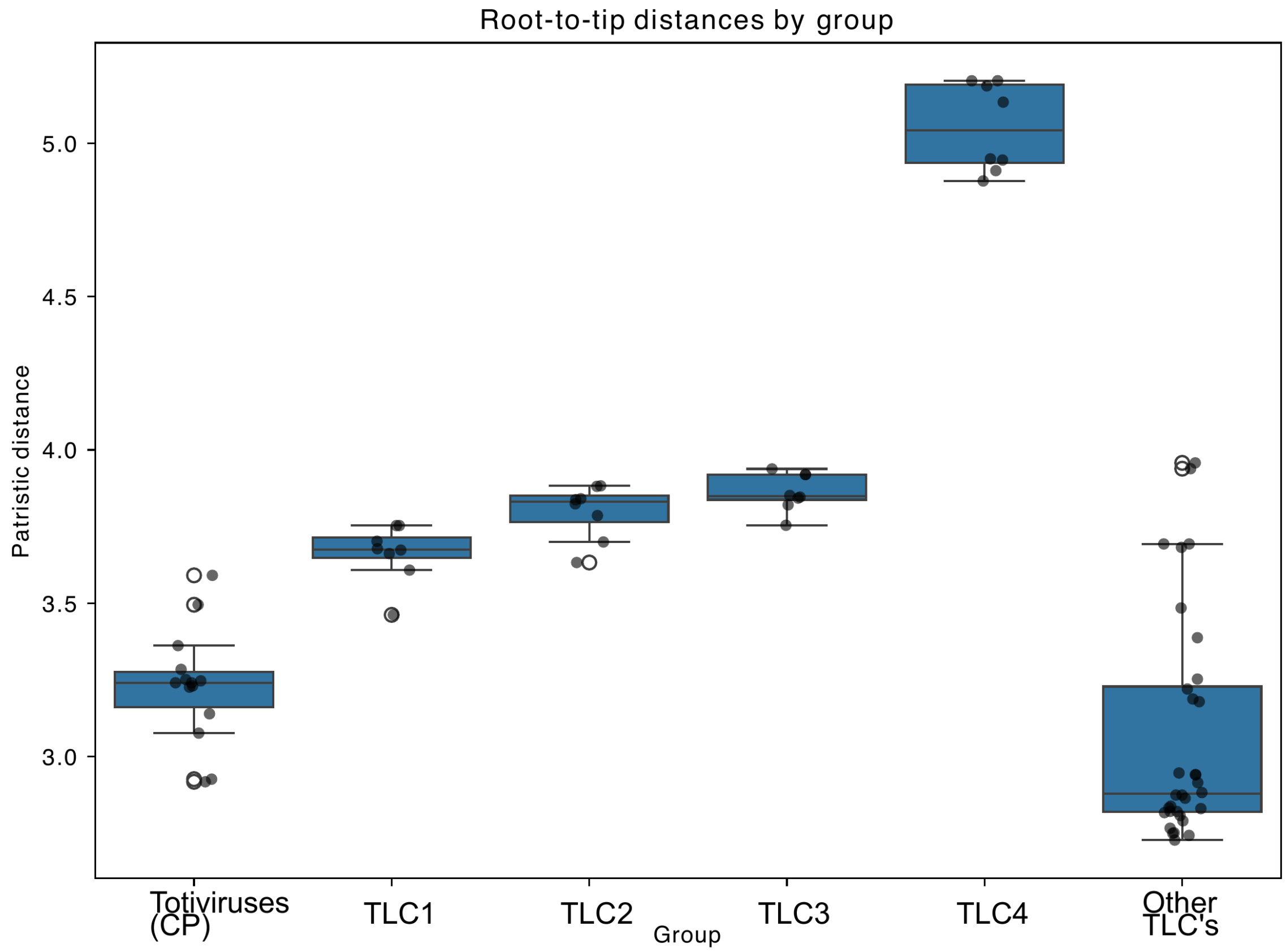

Supplementary Figure S6. **Distribution of sitewise selection signals relative to predicted decapping-associated residues.**

(A) FEL nonsynonymous-rate (β) estimates for eight intact *TLC4* sequences (648 codons), testing all branches of the pruned tree. Light-blue symbols indicate nominal purifying selection ($P \le 0.10$, global BH $q > 0.10$); dark-blue symbols indicate purifying selection after Benjamini-Hochberg correction across all 648 codons ($q \le 0.10$). Red symbols indicate nominal diversifying selection ($P \le 0.10$). Gold stars indicate targeted BH $q \le 0.10$ among five testable, a priori decapping-associated positions; two additional positions were invariant. Open brown circles mark positions summarized in Supplementary Table S8. Upper-boundary triangles indicate $\beta > 4$. Decapping-associated features are numbered within the *TLC4*-only alignment: loop, 150-154; GY-Y, 455; and FAP-F, 558.
(B) Contrast-FEL q-values for the full 658-codon S4 alignment, comparing eight *TLC4* terminal branches with 57 background branches, with a significance threshold of $q \le 0.20$. Corresponding feature positions in this alignment are 153-157, 460, and 564. Shading and dashed vertical lines identify the decapping-associated features.

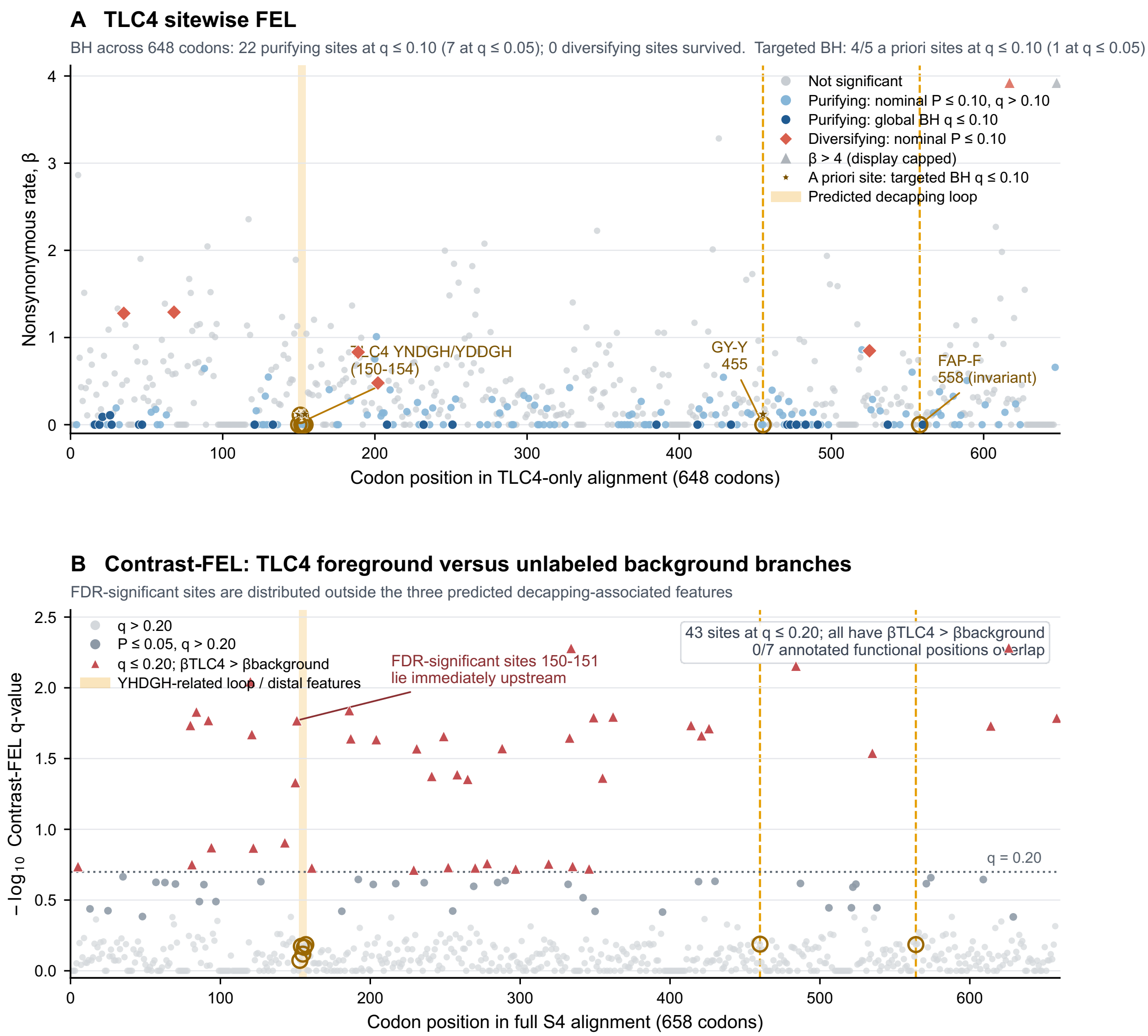

Supplementary Figure S7. **AlphaFold3 prediction confidence for paleoviral capsid structures.**

Violin plots showing the distribution of per-residue pLDDT scores for AlphaFold3 capsid structure predictions across seven structural groups (exogenous totiviruses, TLC1-TLC4 ORFs, TLC4 pseudogenes, singleton paleoviruses). Each point represents one structure (n = 80 total). The dashed line marks pLDDT = 70, the threshold used to define high-confidence residues. All groups showed mean pLDDT > 82 with ≥84% of residues above the threshold, indicating uniformly high prediction quality across structural comparison groups.

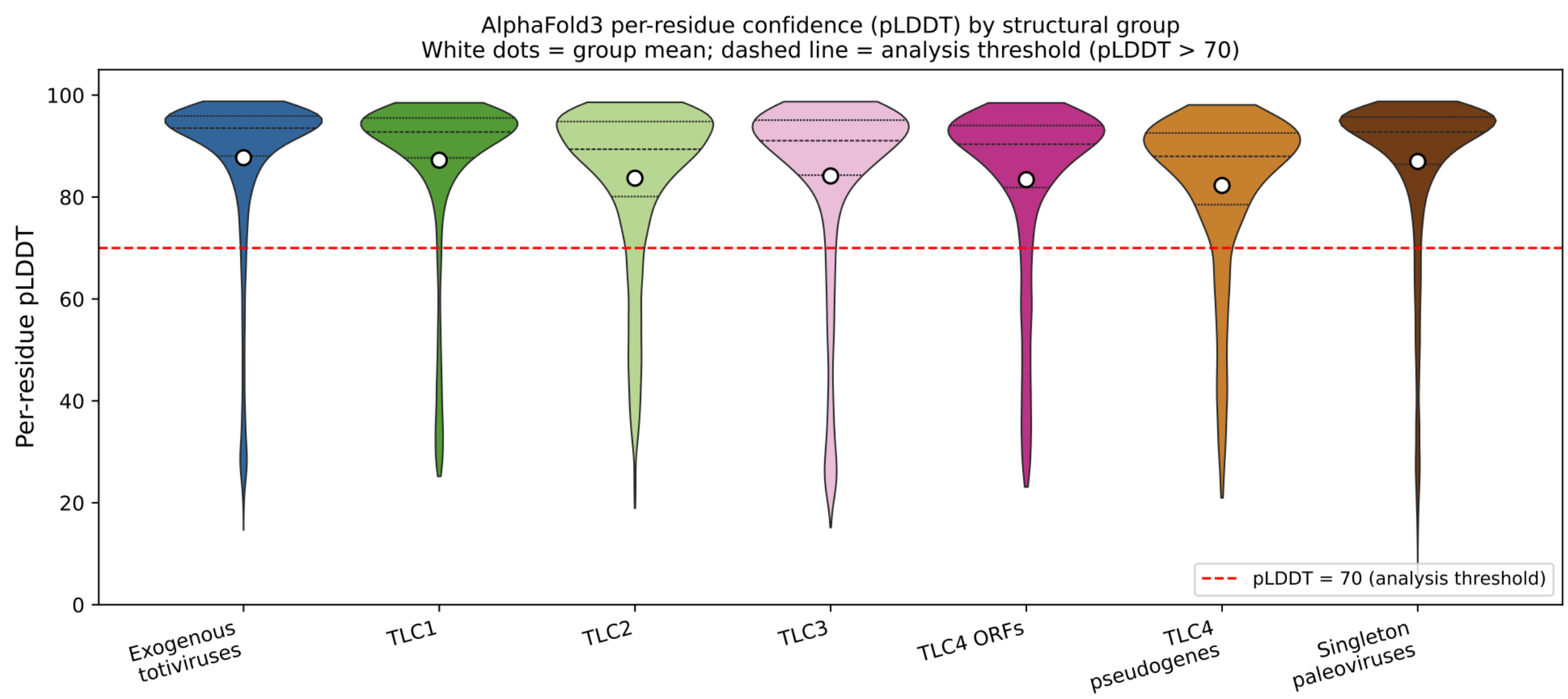

Supplementary Figure S8. PAE heatmaps for representative capsid structures. Predicted Aligned Error (PAE) heatmaps for the highest-confidence representative structure from each of seven structural groups. Lower PAE values (green) indicate high positional confidence; values are shown on a 0-30 Å scale.

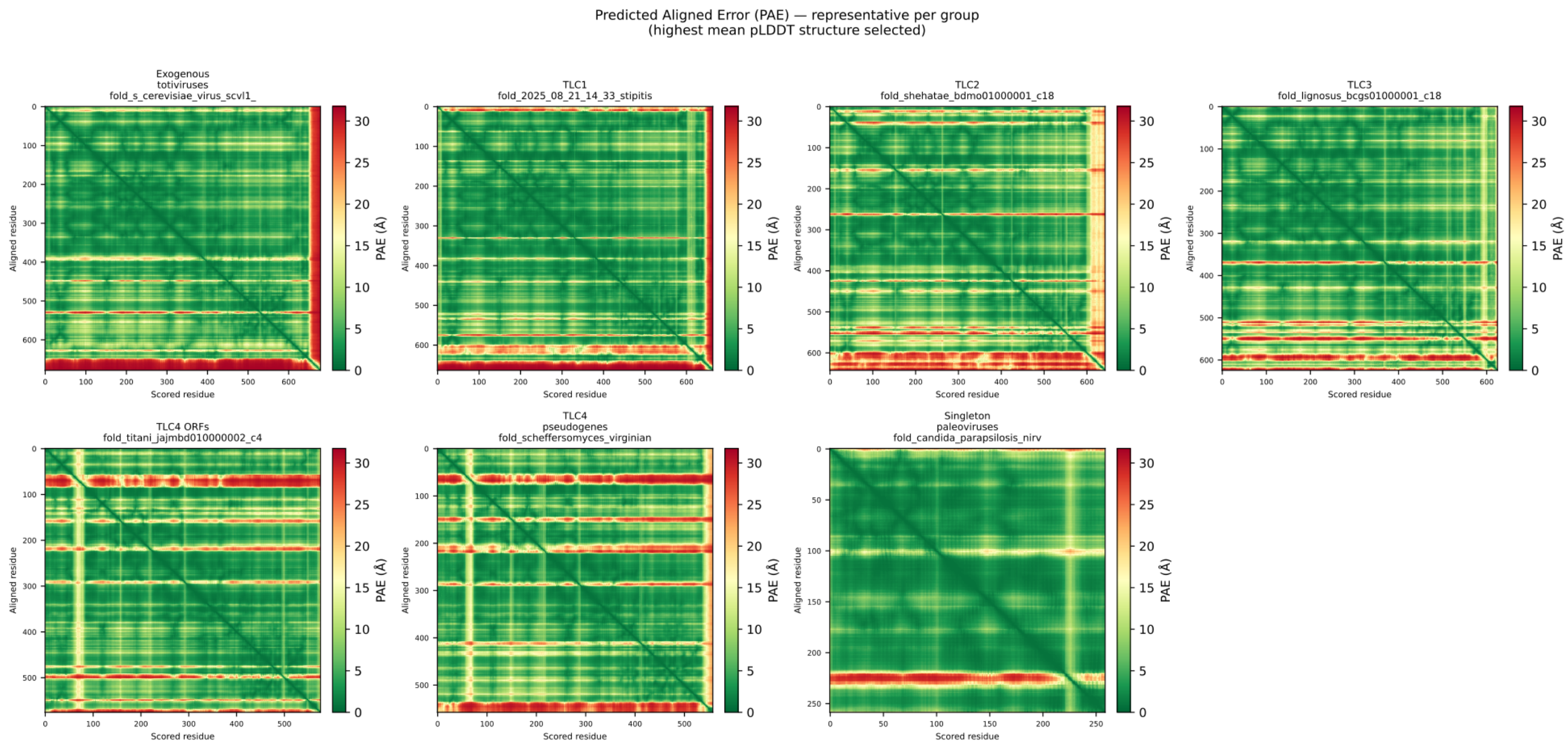

Supplementary Figure S9. PAE heatmaps for all TLC4 ORF structures. PAE heatmaps for all eight TLC4 ORF AlphaFold3 predictions, one per *Scheffersomyces* species/strain. Each panel is labeled by species and genome coordinate.

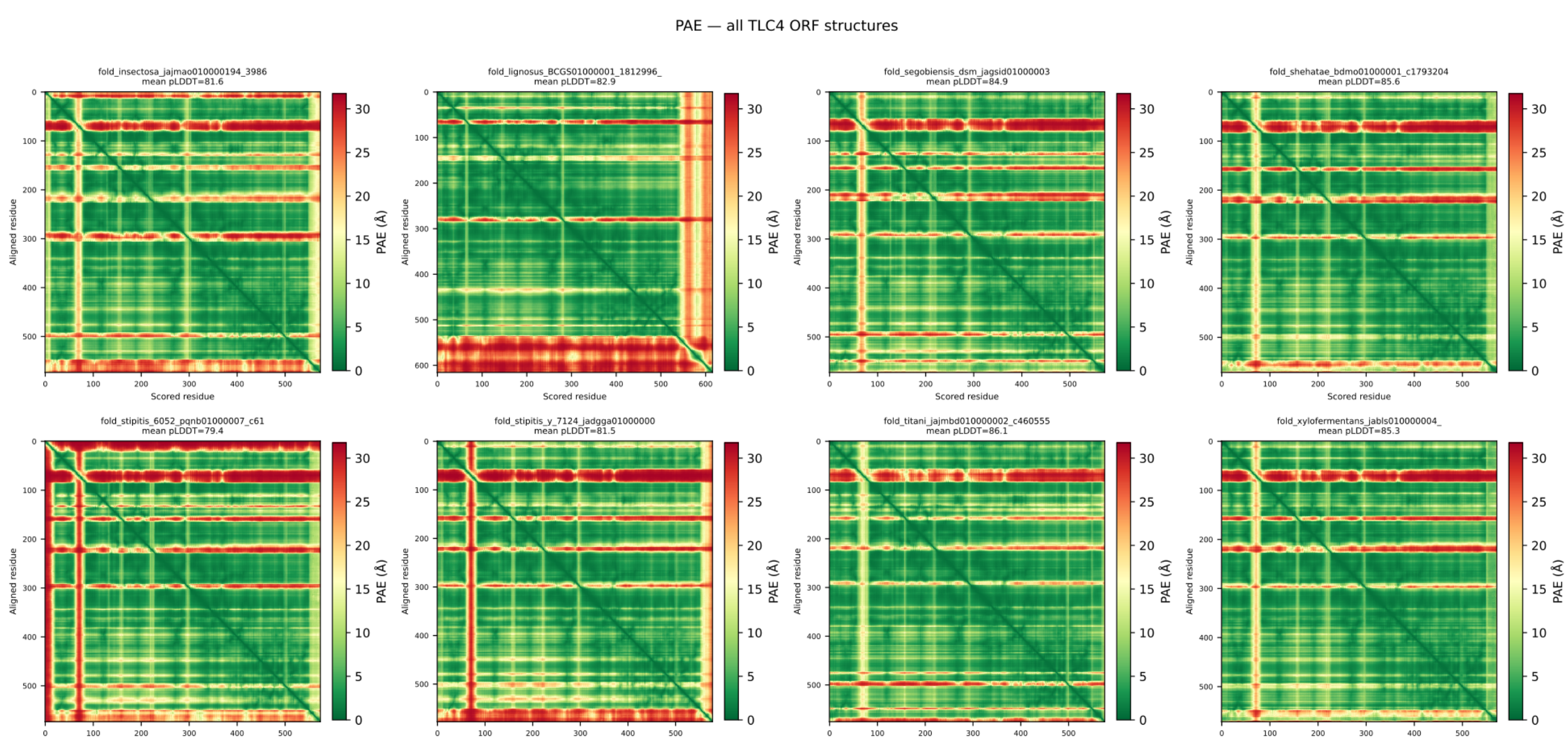

Supplementary Figure S10. Structural similarity of STORM paralogs to an experimentally determined totivirus capsid. AlphaFold3-predicted structures of *Scheffersomyces stipitis* CBS 6054 TLC1 (A), TLC2 (B), TLC3 (C), and TLC4 (D), shown in gold, were aligned to the experimental *Saccharomyces cerevisiae* L-A capsid structure, shown in blue. Foldseek TM-scores are shown below each comparison.

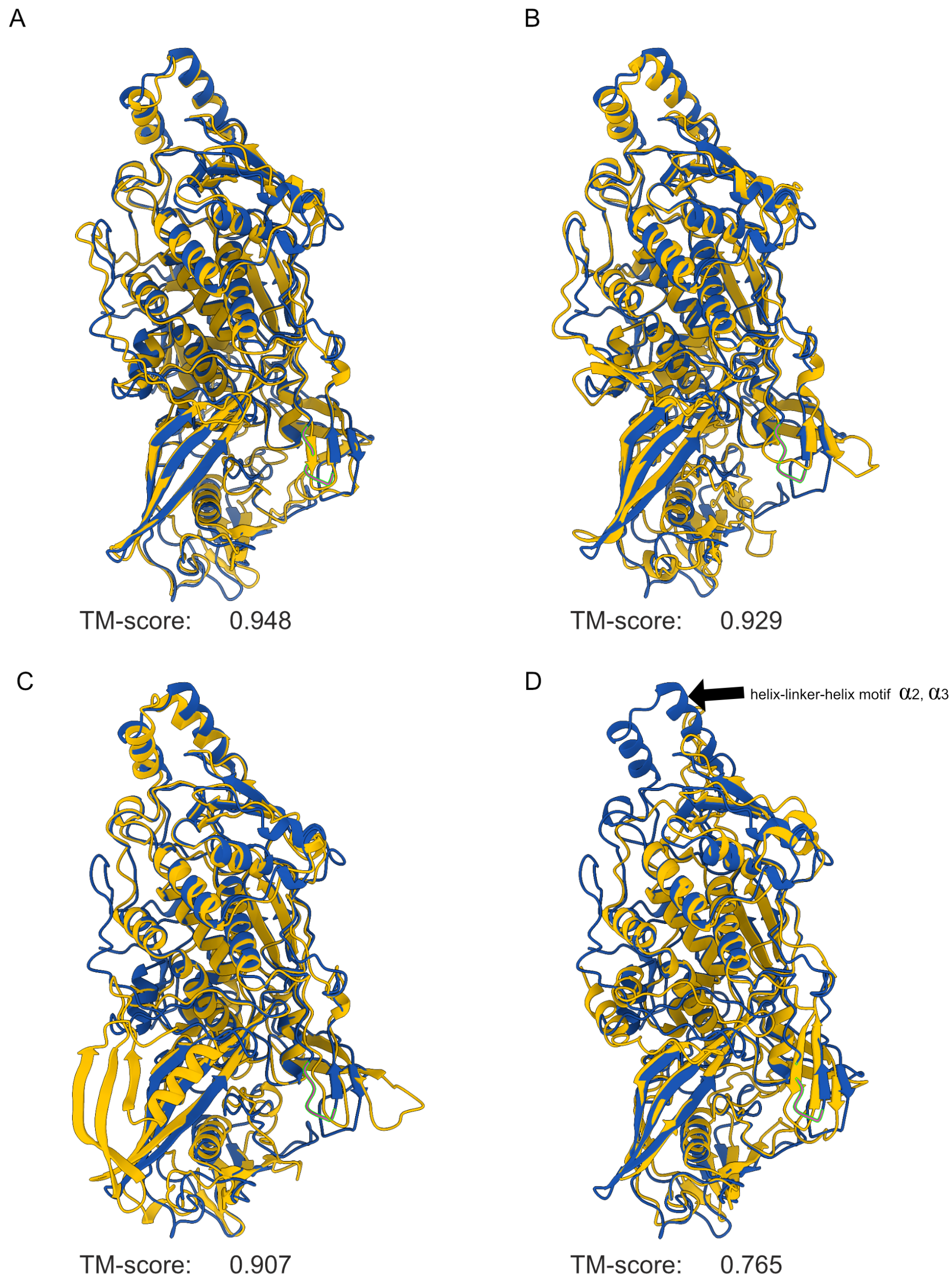

Supplementary Figure S11. Sensitivity of STORM expression estimates to reference normalization. *ACT1*-only $\log_2$ expression contrasts were highly concordant with contrasts scaled to the five-candidate-reference geometric mean (Pearson r = 0.9896; maximum absolute difference = 0.1494 $\log_2$-fold-change units).

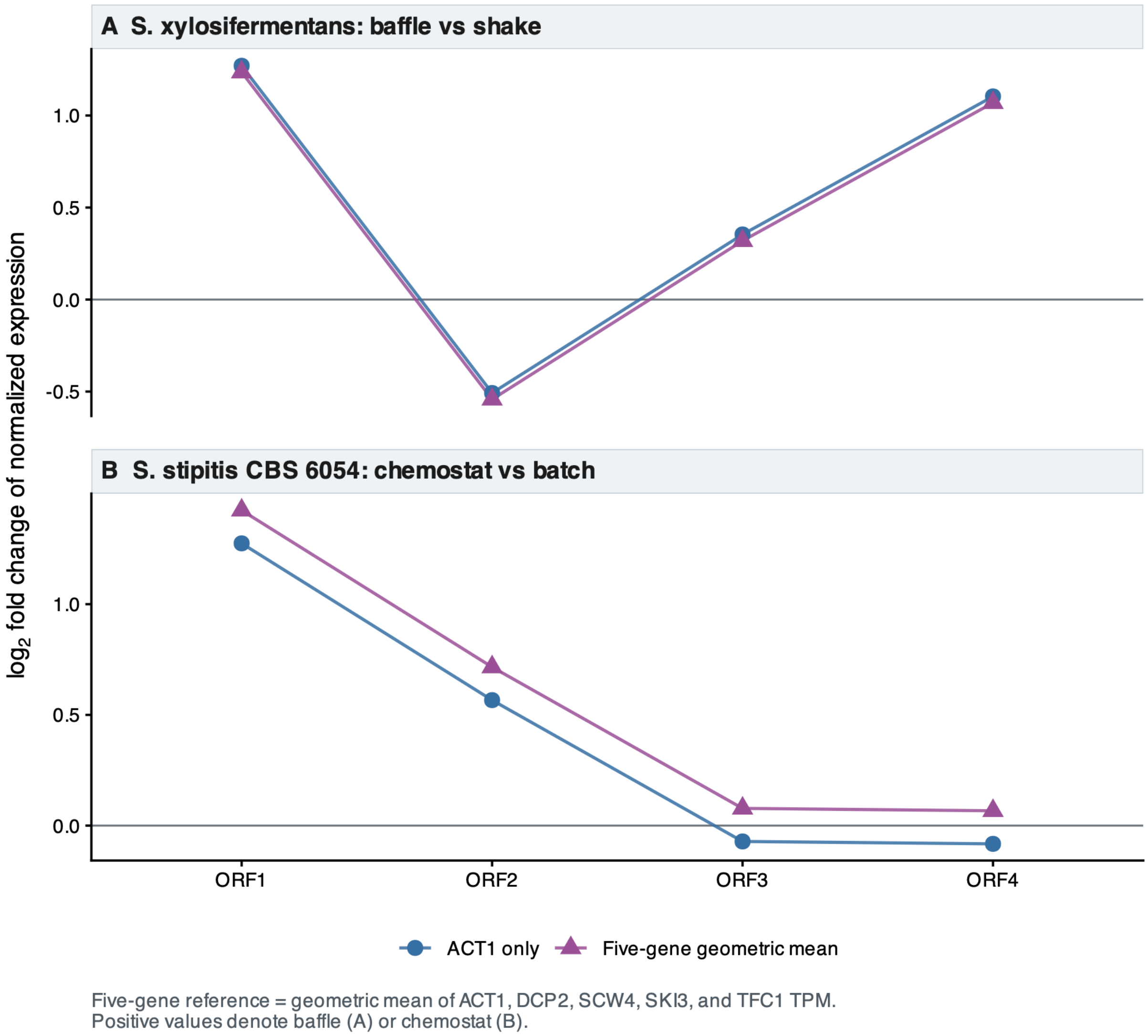

Supplementary Figure S12. Candidate reference genes in replicated differential-expression contrasts. (A) Full-transcriptome differential expression in *S. xylosifermentans* CBS 12540 comparing baffle (higher oxygen transfer and turbulence) versus shake cultures (four biological replicates per condition). The x-axis shows $\log_2$ fold change (baffle/shake; positive values indicate higher expression in baffle), and the y-axis shows $-\log_{10}$ FDR-adjusted *P*-value. **(B)** Full-transcriptome differential expression in *S. stipitis* CBS 6054 comparing chemostat versus batch cultures (two biological replicates per condition; positive values indicate higher expression in chemostat). *ACT1*, *DCP2*, *SCW4*, *SKI3*, and *TFC1* are highlighted and labeled. Vertical dashed lines denote $\log_2$ fold changes of −1 and +1; the horizontal dashed line denotes FDR = 0.05.

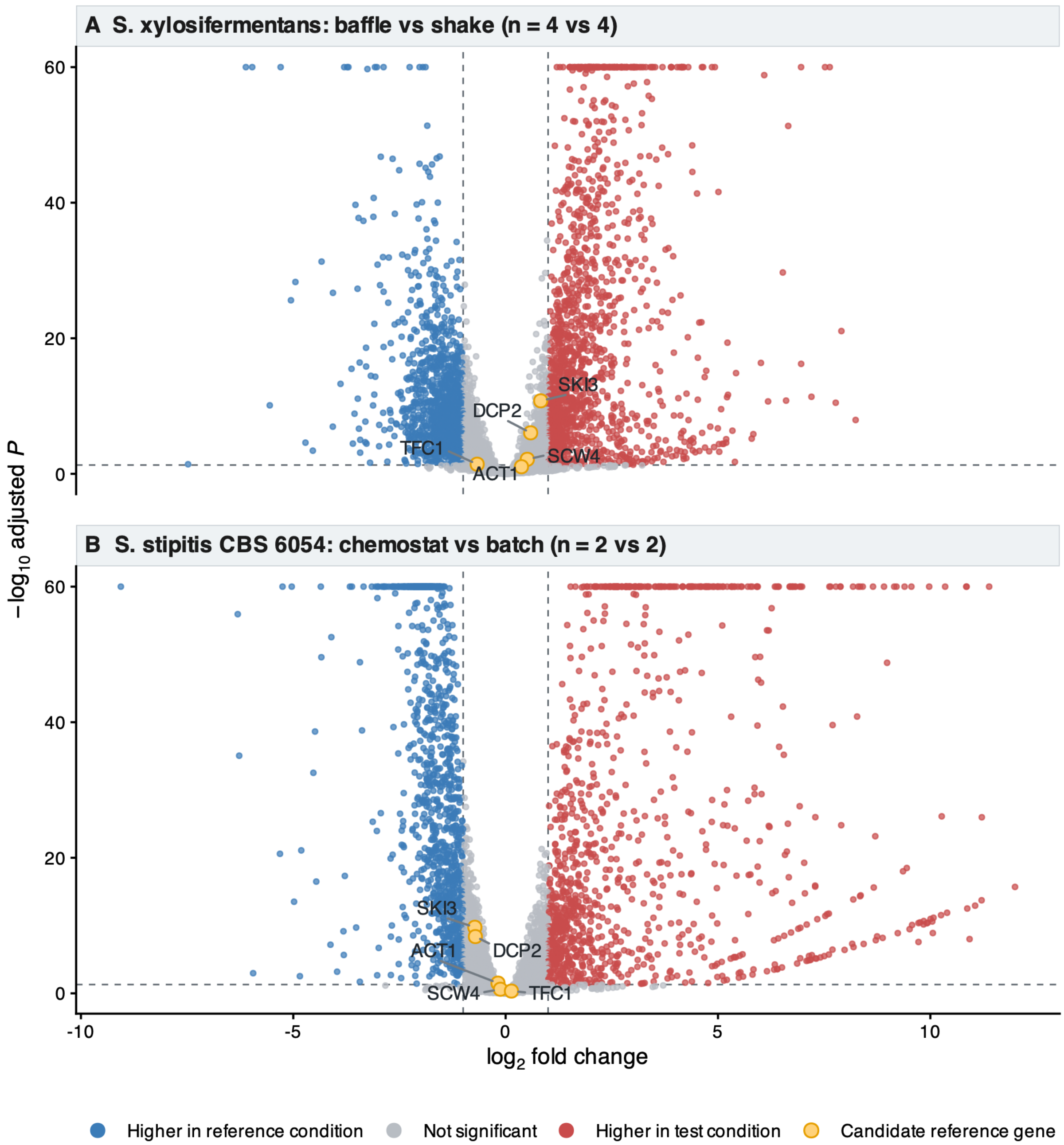

Supplementary Tables

Supplementary Table S1. Genome coordinates of the STORM locus in *Scheffersomyces* species used in microsynteny analysis.

Species	Strain	GenBank Accession	Start (bp)	End (bp)	Span (bp)
Scheffersomyces shehatae	ATY839	BDMO01000001.1	1,775,209	1,815,086	39,878
S. xylosifermentans	CBS 12540	JAXBLS010000004.1	1,678,852	1,719,698	40,847
S. shehatae	NBRC 1983	*Not detected†*	-	-	-
S. virginianus	NRRL Y-48822	JAJMAT010000068.1	1	18,053	18,053
S. quercinus	NRRL Y-48825	JAKTUQ010000029.1	85,000	91,500	6,501
S. parashehatae	CBS 12535	*Not detected†*	-	-	-
S. shehatae	NRRL Y-12858	*Not detected†*	-	-	-
S. cryptocercus	NRRL Y-48824	JANIVO010000022.1	200,000	212,000	12,001
S. insectosa	NRRL Y-12854	JAJMAO010000194.1	1	19,681	19,681
S. lignosus	JCM 9837	BCGS01000001.1	1,801,803	1,834,005	32,203
S. titani	CBS 13926	JAJMBD010000002.1	429,905	472,000	42,096
S. segobiensis	DSM 27193	JAGSID010000034.1	26,295	62,844	36,550
S. segobiensis	NRRL Y-11571	JAKTZB010000011.1	1	16,000	16,000
S. stipitis	CBS 6054	PQNB01000007.1	590,000	628,960	38,961
S. stipitis	NRRL Y-7124	JADGGA010000003.1	1,267,395	1,306,000	38,606
S. illinoinensis	NRRL Y-48827	JAJMAP010000025.1	41,990	62,001	20,012

† Not detected: a syntenic STORM locus could not be identified in available genome assembly for this strain. Coordinates delimit the genomic intervals used for the microsynteny comparisons shown in Fig. 1A.

Supplementary Table S2. YEET element counts by TIR subfamily and transposase clade.

Counts of 213 YEET elements broken down by TIR-sequence-based subfamily (rows) and transposase-phylogeny-based clade (columns). mYEET2b is a non-autonomous MITE (miniature inverted-repeat transposable element) derived from the YEET2b lineage; ? indicates tentative (TIR only) assignment of subfamily. Data are provided in Supplementary Data S1.

TIR subfamily	YEET-α (n)	YEET-β (n)	Total (n)	Median TIR length (bp)	Median element size (bp)	n with TIR / n with size
YEET1a	4	41	45	174	1809	17 / 34
YEET1b	72	23	95	188	1524	45 / 80
YEET1b-Tpase	2	0	2	NA	NA	0 / 0
YEET1c	5	8	13	174	2225	6 / 13
YEET1c?	1	0	1	201	1596	1 / 1
YEET1d	0	2	2	184	1577	2 / 2
YEET1d-like?	0	1	1	NA	NA	0 / 0
YEET1e	2	10	12	195	1791	12 / 12
YEET1f	1	7	8	194	1802	8 / 8
YEET1f-Tpase	0	4	4	NA	NA	0 / 0
YEET1h?	0	1	1	NA	3430	0 / 1
YEET2a	10	0	10	201	1369	10 / 10
YEET2b	1	0	1	201	1941	1 / 1
mYEET2b †	2	0	2	40	1719	1 / 2
YEET-β-*Candida*	0	1	1	NA	2216	0 / 1
unassigned	12	3	15	NA	1535	0 / 5
**Total**	**112**	**101**	**213**			

Supplementary Table S3. Phylogenomic species-tree sensitivity, supported-node tests for the three inferred HGT events, and per-paralog tests of STORM discordance.

Event	Complete STORM/HGT pattern	Partial pattern	Complete species pattern	Weak/other
HGT1	0	0	2,956	571
HGT2	0	3	16	3,508
HGT3	0	0	1,728	1,799

(A) Counts use the strict 3,527-locus, 18-taxon dataset. Event (HGT)-defining nodes were counted only when the corresponding original IQ-TREE edge had SH-aLRT ≥ 80 and ultrafast bootstrap ≥ 95. Complete HGT1 and HGT2 matches required both nested event nodes; HGT3 required the *S. stipitis* NRRL Y-7124 plus *S. segobiensis* node while preserving the broader titani clade. Complete matches also required a strongly supported *S. ergatensis*/*S. coipomoensis* outgroup, and HGT2 additionally required the *S. xylosifermentans*/*S. shehatae* ATY 839 pair. “Partial” indicates that only one of the two HGT2 nodes was recovered; trees lacking required support were classified as weak/other.

Paralog	Sequences	Alignment sites	Best-fit model	lnL, STORM topology	lnL, species-tree topology	ΔlnL	$P_{(AU)}$, species-tree topology	Interpretation
TLC1	12	956	Q.YEAST+G4	−8339.814	−8477.874	138.060	1.75×10^{-8}	Rejected
TLC2	10	956	Q.PLANT+G4	−8246.050	−8356.516	110.467	1.03×10^{-54}	Rejected
TLC3	10	956	Q.PLANT+G4	−8171.882	−8227.736	55.854	1.59×10^{-4}	Rejected
TLC4	13	956	Q.PLANT+G4	−10942.122	−11062.293	120.171	1.12×10^{-4}	Rejected

(B) AU tests of the species-tree-constrained topology. Separate amino-acid alignments were analyzed for each STORM paralog. For each alignment, the maximum-likelihood STORM topology was compared with the corresponding topology constrained to follow the *Scheffersomyces* species tree. The species-tree topology was excluded from the 95% confidence set for every paralog.

Supplementary Table S4. Group descriptive statistics for capsid root-to-tip patristic distances (Figure 5A).

Mean and standard deviation of root-to-tip distances (expected amino-acid substitutions per site) computed from the maximum-likelihood capsid tree underlying Figure 5A. These are descriptive divergence measures. Pairwise tests are in Supplementary Table S5.

Group	Identity	n	Mean root-to-tip	SD
**1**	Exogenous viruses	14	3.23	0.185
**2**	STORM TLC1	8	3.661	0.094
**3**	STORM TLC2	8	3.798	0.089
**4**	STORM TLC3	8	3.862	0.062
**5**	STORM TLC4	8	5.051	0.143
**6**	Non-STORM paleoviruses (Other NIRVs)	32	3.069	0.372

Supplementary Table S5. Pairwise Mann-Whitney U tests of capsid patristic distances between the groups listed in Supplementary Table S4.

Distances were extracted from the maximum-likelihood capsid amino-acid phylogeny (see Methods). Two-sided U statistics and raw *P*-values are reported alongside Benjamini-Hochberg FDR-corrected *P*-values across all 15 comparisons. Sample sizes: exogenous viruses (n = 14), TLC1-TLC4 (n = 8 each), Other NIRVs (non-STORM totivirus-like capsid paleoviruses; n = 32).

Group A	Group B	U	P (raw)	P (FDR)	Sig.
Exogenous viruses	TLC1	2	2.50e-05	9.38e-05	Yes
Exogenous viruses	TLC2	0	6.25e-06	4.69e-05	Yes
Exogenous viruses	TLC3	0	1.51e-04	3.05e-04	Yes
Exogenous viruses	TLC4	0	6.25e-06	4.69e-05	Yes
Exogenous viruses	Other NIRVs	326	0.0154	0.0165	Yes
TLC1	TLC2	9	0.0148	0.0165	Yes
TLC1	TLC3	0	9.31e-04	1.27e-03	Yes
TLC1	TLC4	0	1.55e-04	3.05e-04	Yes
TLC1	Other NIRVs	224	1.24e-03	1.55e-03	Yes
TLC2	TLC3	17	0.128	0.128	*n.s.*
TLC2	TLC4	0	1.55e-04	3.05e-04	Yes
TLC2	Other NIRVs	237	2.43e-04	4.06e-04	Yes
TLC3	TLC4	0	9.31e-04	1.27e-03	Yes
TLC3	Other NIRVs	240	1.63e-04	3.05e-04	Yes
TLC4	Other NIRVs	256	1.62e-05	8.10e-05	Yes

Supplementary Table S6. Host-calibrated PAML local-clock estimates and substitution-model sensitivity.

(A) STORM age with the 97 Ma constraint. Q.YEAST+Γ4 estimates for three STORM crown ages, with the MRCA of Torulaspora delbrueckii virus and the six sampled Saccharomyces L-A viruses fixed at 97 Ma.

Topology	STORM crown (Ma)	Background rate	STORM rate	STORM/background	2Δln L	P
Main	43.2	0.003915	0.034422	8.792	1613.992	6.67×10^{-353}
Main	54.0	0.003951	0.027574	6.979	1252.862	1.98×10^{-274}
Main	65.1	0.003982	0.022910	5.754	998.923	3.08×10^{-219}
Alternative	43.2	0.003810	0.034001	8.925	1731.134	2.35×10^{-378}
Alternative	54.0	0.003859	0.027216	7.053	1327.973	9.43×10^{-291}
Alternative	65.1	0.003907	0.022594	5.782	1037.759	1.11×10^{-227}

(B) Sensitivity to a deeper host-based calibration. The 97 Ma constraint was replaced by an age assigned to the MRCA of Lodderomyces beijingensis virus and the sampled Saccharomyces L-A lineage. All nine combinations of three STORM ages and the deeper host-node mean (235.0521 Ma) and 95% credible-interval bounds (198.455-277.600 Ma; Shen et al. 2018) were evaluated under Q.YEAST+Γ4. The Torulaspora-Saccharomyces viral node age was estimated rather than fixed.

Topology	STORM crown (Ma)	Deeper node (Ma)	Background rate	STORM rate	STORM/background	2Δln L	P
Main	43.2	198.5	0.004383	0.034452	7.861	1373.122	1.46×10^{-300}
Main	54.0	198.5	0.004406	0.027604	6.265	1172.694	5.24×10^{-257}
Main	65.1	198.5	0.004431	0.022939	5.177	996.059	1.29×10^{-218}
Main	43.2	235.1	0.003688	0.034423	9.334	1515.200	1.95×10^{-331}
Main	54.0	235.1	0.003705	0.027571	7.442	1325.961	2.58×10^{-290}
Main	65.1	235.1	0.003722	0.022901	6.153	1156.243	1.97×10^{-253}
Main	43.2	277.6	0.003114	0.034400	11.047	1644.872	1.30×10^{-359}
Main	54.0	277.6	0.003126	0.027545	8.812	1468.622	2.58×10^{-321}
Main	65.1	277.6	0.003138	0.022873	7.288	1307.746	2.34×10^{-286}
Alternative	43.2	198.5	0.004239	0.034019	8.024	1899.362	6.63×10^{-415}
Alternative	54.0	198.5	0.004268	0.027234	6.381	1574.034	3.21×10^{-344}
Alternative	65.1	198.5	0.004299	0.022611	5.260	1299.653	1.34×10^{-284}
Alternative	43.2	235.1	0.003565	0.034008	9.540	2135.884	2.73×10^{-466}
Alternative	54.0	235.1	0.003585	0.027219	7.593	1821.755	4.81×10^{-398}
Alternative	65.1	235.1	0.003606	0.022592	6.265	1547.962	1.48×10^{-338}
Alternative	43.2	277.6	0.003008	0.034001	11.302	2354.100	1.07×10^{-513}
Alternative	54.0	277.6	0.003022	0.027209	9.003	2057.963	2.31×10^{-449}
Alternative	65.1	277.6	0.003037	0.022579	7.435	1791.927	1.46×10^{-391}

Supplementary Table S6 (continued).

(C) Substitution-model sensitivity at the central calibrations. Q.YEAST+Γ4 and JTT+F+Γ4 were compared at a STORM crown age of 54 Ma under the 97-Ma and 235.0521-Ma calibration schemes. Q.YEAST used its published amino-acid frequencies; JTT+F used frequencies from the alignment.

Model	Topology	Viral node (Ma)	Background rate	STORM rate	STORM/background	2Δln L	P
Q.YEAST+Γ4	Main	97.0	0.003951	0.027574	6.979	1252.862	1.98×10^{-274}
JTT+F+Γ4	Main	97.0	0.003748	0.024907	6.645	1247.894	2.38×10^{-273}
Q.YEAST+Γ4	Alternative	97.0	0.003859	0.027216	7.053	1327.973	9.43×10^{-291}
JTT+F+Γ4	Alternative	97.0	0.003664	0.024587	6.710	1322.655	1.35×10^{-289}
Q.YEAST+Γ4	Main	235.1	0.003705	0.027571	7.442	1325.961	2.58×10^{-290}
JTT+F+Γ4	Main	235.1	0.003471	0.024894	7.172	1343.850	3.34×10^{-294}
Q.YEAST+Γ4	Alternative	235.1	0.003585	0.027219	7.593	1821.755	4.81×10^{-398}
JTT+F+Γ4	Alternative	235.1	0.003365	0.024582	7.304	1843.159	1.08×10^{-402}

Notes: All analyses used the same 46-sequence, 887-position capsid alignment. H_0 fitted one rate to all branches; H_1 fitted separate rates for branches within the STORM crown and all remaining branches, including the incoming STORM stem and Black raspberry virus F. Rates are H_1 estimates in expected amino-acid substitutions per site per million years. Likelihood-ratio tests used one degree of freedom.

Supplementary Table S7. RELAX tests of selection intensity (Wertheim et al. 2015) on *Scheffersomyces* STORM capsid paralogs.

The RELAX test estimates a relaxation/intensification parameter K relative to a reference branch set, where K < 1 indicates relaxation and K > 1 intensification. LR, likelihood-ratio statistic; P, associated P-value (df = 1). Analyses used HyPhy and the Alternative Yeast Nuclear code (NCBI Table 12). STORM, *Scheffersomyces* Totivirus-like ORF Repeat Module; TLC, Totivirus-Like Capsid.

Comparison	Test branch set	Reference branch set	K	LR	*P*	Interpretation
STORM *TLCs* vs. exogenous viruses	All STORM capsid branches	Exogenous totivirus capsid branches	0.78	3.16	0.075	Non-significant trend toward relaxation
TLC1 vs. *TLC2+TLC3*	*TLC1* branches	*TLC2* and *TLC3* branches	1.74	16.47	<0.001	Significant intensification of purifying selection on *TLC1*
TLC4 vs. other STORM *TLCs*	*TLC4* branches	Other STORM TLC branches (TLC1-TLC3)	0.29	74.13	<0.001	Strong, significant relaxation of selection on *TLC4*

Supplementary Table S8. Sitewise FEL estimates at predicted decapping-associated residues in *TLC4* and homologous positions in *TLC1*/2/3.

S4 position	Motif	*TLC4* codon variation	*TLC4* β	*TLC4* P / targeted q	*TLC1*/2/3 β	*TLC1*/2/3 P / targeted q
153	YHDGH - Y	synonymous (tat/tac)	0.000	0.0666 / 0.0941	0.051	0.00174 / 0.00608
154	YHDGH - H	N↔D (conservative)	0.108	0.5981 / 0.5981	0.268	0.4399 / 0.4399
155	YHDGH - D	invariant (gac×8)	-	invariant	0.144	0.2265 / 0.2643
156	YHDGH - G	synonymous (ggg/gga)	0.000	0.00443 / 0.0221	0.273	0.0125 / 0.0292
157	YHDGH - H	synonymous (cac/cat)	0.000	0.0472 / 0.0941	0.231	0.0381 / 0.0667
460	GY - Y	synonymous (tac/tat)	0.000	0.0753 / 0.0941	0.377	0.00152 / 0.00608
564	FAP - F	invariant (ttt×8)	-	invariant	0.253	0.1055 / 0.1477

Site-specific β (nonsynonymous rate), raw P-values, and targeted BH q-values from FEL analyses of *TLC4* (8 sequences; 648-codon *TLC4*-only alignment) and *TLC1*/2/3 (24 sequences; 658-codon S4 alignment). P / q entries give the raw P-value followed by the Benjamini-Hochberg q-value for the prespecified decapping-site family. Positions are numbered according to the S4 reference alignment (658 codons).

Supplementary Table S9. Reference nuclear genes used for species-tree inference and introgression testing in *Scheffersomyces*.

Accession numbers are from *S. stipitis* CBS 6054 (GCF_000209165.1) except KC616421.1 (*ELP3*). Gene product annotations are from UniProt/SGD. The same gene panel was used in Aguileta et al. (2008) and Taylor et al. (2013).

Accession	Gene	Gene product / function
XM_001384142.1	*MCM7*	Mini-chromosome maintenance complex component 7
KC616421.1	*ELP3*	Elongator complex protein 3
XM_001387404.1	*KOG1*	TORC1 subunit Kog1 (Raptor homolog)
XM_001384848.1	*GDH2*	NAD-dependent glutamate dehydrogenase
XM_001384577.1	*ILV2*	Acetolactate synthase, large subunit
XM_001382598.1	*UBA1*	Ubiquitin-activating enzyme E1 1
XM_001386823.1	*POL1*	DNA polymerase alpha, catalytic subunit
XM_001387653.1	*RBV1*	Protein Rbv1 (Ribosome biogenesis)
XM_001384154.1	*MSH3*	DNA mismatch repair protein Msh3
XM_001387301.1	*CCT8*	T-complex protein 1 subunit theta (Cct8)

Supplementary Table S10. Public RNA-seq datasets used for targeted STORM expression analyses.

RNA-seq samples used for descriptive transcription estimates and replicated DESeq2 contrasts. Differential-expression inference was restricted to four baffle and four shake biological replicates for *S. xylosifermentans* CBS 12540 and two batch and two chemostat replicates for *S. stipitis* CBS 6054. Single-sample datasets were used only as descriptive evidence of transcription. Complete count matrices, DESeq2 outputs, metadata, scripts, and validation are in Supplementary Data S9.

Species	Strain	Source	Condition	Rep.	n/condition	DE inference	Contrast/use
S. insectosa	NRRL Y-12854	JGI MycoCosm: Mycocosm_AP-1175634	single; condition unavailable	1	1	No	descriptive only
S. lignosus	JCM 9837(=NRRL Y-12856)	JGI MycoCosm: Mycocosm_AP-1145561	single; condition unavailable	1	1	No	descriptive only
S. stipitis	CBS 6054	SRR453573	batch	1	2	Yes	chemostat/batch
S. stipitis	CBS 6054	SRR453574	batch	2	2	Yes	chemostat/batch
S. stipitis	CBS 6054	SRR453576	chemostat	1	2	Yes	chemostat/batch
S. stipitis	CBS 6054	SRR453577	chemostat	2	2	Yes	chemostat/batch
S. stipitis	NRRL Y-7124	SRR8420582	single public sample	1	1	No	descriptive only
S. xylosifermentans	CBS 12540	SRR28362661	baffle	1	4	Yes	baffle/shake
S. xylosifermentans	CBS 12540	SRR28362662	baffle	2	4	Yes	baffle/shake
S. xylosifermentans	CBS 12540	SRR28362663	baffle	3	4	Yes	baffle/shake
S. xylosifermentans	CBS 12540	SRR28362664	baffle	4	4	Yes	baffle/shake
S. xylosifermentans	CBS 12540	SRR28362665	shake	1	4	Yes	baffle/shake
S. xylosifermentans	CBS 12540	SRR28362666	shake	2	4	Yes	baffle/shake
S. xylosifermentans	CBS 12540	SRR28362667	shake	3	4	Yes	baffle/shake
S. xylosifermentans	CBS 12540	SRR28362668	shake	4	4	Yes	baffle/shake
S. xylosifermentans	CBS 12540	SRR26057920	single xylose culture	1	1	No	descriptive only

Supplementary Data

Supplementary Data S1. YEET (Yeast Endogenous Element Transposon) element data file :Supplementary_Data_S1_YEET_elements.xlsx.

Supplementary Data S2. Metadata for the 84-taxon capsid amino-acid alignment underlying Figures 2 and 5A: CP_MAFFT_84_taxa_supplement.xlsx.

Supplementary Data S3. Capsid amino-acid alignment underlying Figures 2 and 5A.

Supplementary Data S4. Capsid nucleotide alignment of *Scheffersomyces* TLC paralogs with two outgroups (Figure 3).

Supplementary Data S5. RNA-dependent RNA polymerase amino-acid alignment underlying Figure 5C and Supplementary Figure S2.

Supplementary Data S6. YEET transposase amino-acid alignment (used for Supplementary Figure S3).

Supplementary Data S7. AlphaFold3 predicted capsid structures and Foldseek/pLDDT/PAE outputs underlying Figure 6 and Supplementary Figures S7-S10.

Zip archive (Supplementary_Data_S7_AlphaFold3_predictions.zip), archived at Zenodo (doi: 10.5281/zenodo.22900571).

Supplementary Data S8. Phylogenetic concordance and patristic-distance summaries underlying Figure 4. MAFFT alignments and IQ-TREE outputs are deposited on Zenodo (doi: 10.5281/zenodo.22900571).

Supplementary Data S9. Expression analysis archive containing complete raw and DESeq2-normalized count matrices, full DESeq2 result tables, sample metadata, reference-gene validation, analysis scripts, session information, and README.

Supplementary Data S10. Expanded phylogenomic analysis containing OrthoFinder orthogroup QC, primary and strict IQ-TREE gene-tree sets (STORM excluded), ASTRAL sensitivity trees and summary, event-specific full-tree supported-node classifications and counts, scripts, and README.

References

Aguileta G, Marthey S, Chiapello H, Lebrun M-H, Rodolphe F, Fournier E, Gendrault-Jacquemard A, Giraud T. 2008. Assessing the Performance of Single-Copy Genes for Recovering Robust Phylogenies. Systematic Biology 57:613-627.

Barros KO, Mader M, Krause DJ, Pangilinan J, Andreopoulos B, Lipzen A, Mondo SJ, Grigoriev IV, Rosa CA, Sato TK, Hittinger CT. 2024. Oxygenation influences xylose fermentation and gene expression in the yeast genera *Spathaspora* and *Scheffersomyces*. Biotechnology for Biofuels and Bioproducts 17:20. doi:10.1186/s13068-024-02467-8.

Papini M, Nookaew I, Uhlén M, Nielsen J. 2012. *Scheffersomyces stipitis*: a comparative systems biology study with the Crabtree positive yeast *Saccharomyces cerevisiae*. Microbial Cell Factories 11:136. doi:10.1186/1475-2859-11-136.

Taylor DJ, Ballinger MJ, Bowman SM, Bruenn JA. 2013. Virus-host co-evolution under a modified nuclear genetic code. PeerJ 1:e50.

Wertheim JO, Murrell B, Smith MD, Kosakovsky Pond SL, Scheffler K. 2015. RELAX: detecting relaxed selection in a phylogenetic framework. Mol Biol Evol 32:820-832.
